# Genome-wide association study of pre-harvest sprouting resistance and grain color in common wheat (*Triticum aestivum* L.)

**DOI:** 10.1186/s12870-025-07039-y

**Published:** 2025-07-29

**Authors:** Ling Chen, Yue Tao, Chengxiang Song, Yike Liu, Hanwen Tong, Qiang Ning, Juan Zou, Penghao Fu, Yuqing Zhang, Chunbao Gao, Zhanwang Zhu

**Affiliations:** 1 Institute of Food Crops, Hubei Academy of Agricultural Sciences/Central China Wheat Disease Biology Research Station of Ministry of Agriculture and Rural Affairs/Hubei Engineering and Technology Research Center of Wheat/Hubei Key Laboratory of Food Crop Germplasm and Genetic Improvement/Key Laboratory of Crop Molecular Breeding of Ministry of Agriculture and Rural Affairs, Wuhan, Hubei 430064 China; 2 National Key Laboratory of Crop Genetic Improvement, Huazhong Agricultural University, Wuhan, Hubei 430070 China

**Keywords:** Wheat, PHS resistance, Grain color, GWAS, QTLs, KASP markers

## Abstract

**Background:**

Pre-harvest sprouting (PHS) is a serious problem in wheat production globally. Grain color (GC) has a notable impact on PHS resistance, red grains typically show higher resistance compared to white grains. To understand the genetic factors influencing PHS and GC, a genome-wide association study (GWAS) was conducted on a natural population of 235 wheat cultivars using a 90 K single nucleotide polymorphism (SNP) arrays.

**Results:**

A strong correlation between PHS and GC was observed, with the highest correlation coefficient of 0.85 (*P* < 0.0001). Association mapping was performed using four different models (BLINK, FarmCPU, MMLM and MLM) in the GAPIT along with MLM model in the Tassel. The study identified twelve stable quantitative trait loci (QTLs) related to PHS resistance and another twelve stable QTLs associated with GC. Notably, six QTLs for PHS resistance were newly discovered, explaining 5.8–10.0% of the phenotypic variation. Additionally, four common QTLs were identified that are linked to both PHS resistance and GC. Among these, *Qphs.hbaas-3B.2*/*Qgc.hbaas-3B.2* and *Qphs.hbaas-3D*/*Qgc.hbaas-3D* were recognized as major loci significantly affecting both traits, likely associated with the genes *Tamyb-B1* and *Tamyb-D1*, respectively. The other two new QTLs on chromosome 2B explained 7.0–10.0% of phenotypic variation in PHS resistance and 4.7–7.4% of phenotypic variation in GC. Furthermore, six candidate genes associated with PHS resistance were predicted, warranting further investigation. Three KASP markers *IACX5850*, *Tdurum_contig11028_236* and *wsnp_Ex_c269_518324* linked to three QTLs (*Qphs.hbaas-2B.2*, *Qphs.hbaas-2B.4*, and *Qphs.hbaas-7B.2*) are applicable for marker-assisted selection in wheat breeding to enhance PHS resistance.

**Conclusions:**

This study provides valuable genetic loci and KASP markers that can enhance PHS resistance in wheat breeding programs and offers insights for discovering PHS resistance genes.

**Supplementary Information:**

The online version contains supplementary material available at 10.1186/s12870-025-07039-y.

## Background

Wheat PHS occurs when grains sprout prematurely in spikes before they are harvested, primarily due to broken dormancy triggered by sustained rainfall. Although there may be no visible signs of germination, starch and protein degradation takes place, leading to reductions in both grain yield and quality. PHS presents a significant challenge to wheat production, resulting in economic losses throughout the industry [1]. Regions around the world that grow wheat, including China, Japan, the USA, Canada, Europe, South Africa, Australia, and others, have reported instances of PHS [2]. Given the ongoing challenges posed by global climate change, it is crucial to address and mitigate the risks associated with PHS.

Developing wheat varieties that are resistant to PHS is an effective strategy to reduce the damage caused by this issue. PHS resistance is a quantitative trait influenced by multiple genes. To date, many QTLs associated with PHS resistance have been identified across all 21 wheat chromosomes [2]. Several major QTLs have been found on chromosomes 3 A [3–5], 3B [4, 6], 3D [7, 8], and 4 A [4, 5]. However, only a limited number of genes that confer PHS resistance have been identified. These include *TaPHS1/TaMFT* [9], *TaMKK3* [10], *TaSdr* [11], *TaVp1* [12], *Tamyb10* [13], and *TaQsd1* [14]. *TaPHS1*, a key gene underlying the 3AS QTL, has been shown to account for up to 58% of the phenotypic variation in PHS resistance [15]. *TaMKK3*, causal for *Phs1-4AL*, explained up to 43.3% of the phenotypic variation [16]. Pyramiding these QTLs and associated genes could further improve resilience against PHS in wheat.

Seed dormancy, pivotal to PHS resistance, is modulated by environment, phytohormones, and molecular factors. Abscisic acid (ABA) and gibberellic acid (GA) act antagonistically to regulate seed dormancy and germination. Other hormones, such as auxin/indole-3-acetic acid (IAA), brassinosteroid (BR), jasmonate (JA), ethylene (ET), and salicylic acid (SA), also affect dormancy [2]. In addition to phytohormones, many transcription factors have been found to be involved in seed dormancy. For example, *ABI3*, *HSI2/VAL1*, *HSL1/VAL2*, *ABI4*, *CHO1 (CHOTTO1)*, and *ABI5*, each containing unique domains such as B3, APETALA 2 (AP2), and basic leucine zipper (bZIP), regulated dormancy through ABA or GA pathway [17–20]. Additionally, MYB transcription factors, including *Tamyb10* and *NtMYB330*, have been shown to pleiotropically enhance seed dormancy and proanthocyanidin biosynthesis [13, 21]. Moreover, genes related to chromatin remodeling are also involved in the regulation of seed dormancy. For instance, the *RELATIVE OF EARLY FLOWERING6* (*REF6*) gene, which encoding an H3K27me3 demethylase, reduced seed dormancy by promoting ABA catabolism [22]. *RDO2* encoded a transcription elongation factor (TFIIS) that affected seed dormancy by influencing transcription elongation efficiency [23]. The chromatin-remodeling factor *PICKLE* (*PLK*) inhibited seed dormancy by regulating the H3K27me3 levels of *DOG1* expression [24]. By exploring genes associated with these transcription factors and phytohormones within QTLs related to PHS resistance, researchers could uncover new candidate genes that contributed to seed dormancy and PHS tolerance.

Red-grained wheat shows higher PHS resistance than white-grained varieties, indicating a link between GC and PHS resistance, as supported by multiple studies [25–27]. Specifically, a notable correlation coefficient of 0.73 was observed between GC and PHS when assessing GC via a method that involved soaking seeds in sodium hydroxide (NaOH) solution [28]. Furthermore, the use of an a/L value derived from a colorimeter for GC measurement yielded a correlation coefficient of 0.49 with PHS [25]. Our previous research also observed a robust correlation between GC and PHS when utilizing an a/b colorimeter value as the GC trait [29].

In the present study, a GWAS was conducted on 235 elite wheat cultivars to uncover novel QTLs associated with PHS resistance and GC. The objectives were fourfold: (1) to analyze phenotypic variations in PHS and GC; (2) to investigate the correlation between different GC traits and PHS resistance; (3) to detect QTLs linked to PHS resistance and GC; (4) to identify potential candidate genes and KASP markers conferring PHS resistance. These identified markers and candidate genes will serve as valuable resources for breeding wheat with PHS resistance.

## Materials and methods

### Plant materials

A natural population comprising 235 wheat cultivars was used for GWAS (Table S1). This population originated from the panel which was recently analyzed for stripe rust resistance and Fusarium head blight resistance [30, 31]. Wheat cultivars from China were divided into several subgroups based on China’s 10 major agro-ecological production zones [32]. There were 7 cultivars from Zone I, 139 from Zone II, 48 from Zone III, 18 from Zone IV, and 14 from Zone VIII. 9 cultivars from CIMMYT were designated as CIMMYT Zone. All cultivars were planted in a rice field at Nanhu Experimental Station in Wuhan, Hubei Province (114.31672° E, 30.48372° N) across five cropping seasons (2015–2019). Field management followed local agricultural practices throughout the growing period.

### Phenotyping

Spikes were harvested at Zadoks stage 87 (wax ripeness), defined by golden-yellow grain color, stem yellowing, and resistance to fingernail pressure. Ten intact spikes with 10 cm peduncle were collected from each cultivar and separated into two replicates (5 spikes per technical replicate). All spikes were air dried for 7 days at room temperature, avoiding direct sunlight and high temperature, then stored at − 20℃ to maintain dormancy until tests were conducted, within 1–3 months after harvest (Fig. S1).

Spike-wetting tests were conducted in a rain simulation chamber to evaluate PHS tolerance. Spikes from the freezer were placed upright on perforated stainless-steel trays and allowed to naturally air dry at room temperature for 2 days. Each tray could hold 10 rows and 15 columns of spikes, with 3.2 cm separating the spikes. The rain simulation chamber was set to spray water for 20 min every 4 h at 20 ± 2 °C. During the second day, a 0.025 mg mL^–1^ Quintozene solution was sprayed for 5 min to control fungal contamination [33]. To ensure uniformity of the wetting treatment, the trays were repositioned within the chamber at the same time each day. After 5 days of incubation, each germinated spike was put in a parchment bag and stored at − 20℃ until manual counting.

Germinated spikes were threshed by hand. A grain was considered germinated when either the radicle or plumule exceeded half of the grain’s length. The germination percentage (GP) for each year, from 2015 to 2019, was calculated as the percentage of germinated grains to the total number of grains in five spikes of a cultivar. The average GP of two replicates assayed in 2015–2019 was designated GP15, GP16, GP17, GP18, and GP19, respectively (Table S1).

Grain color (GC) was measured by a colorimeter (Konica Minolta CM-5, https://sensing.konicaminolta.asia/product/cm-5-spectrophotometer/) in 2019. Color values *L* (0 to 100, measuring dark to brightness), *a* (–100 to 100, indicating the green to red), and *b*(–100 to 100, representing blue to yellow) were recorded [34]. For each cultivar, the color values *L*, *a*, and *b*, were measured three times, and their mean values were used. Wheat grains were also visually estimated as white-grained (assigned a value of 1) or red-grained (assigned a value of 2). After correlation analysis between colorimeter values (*L*, *a*, *b*, *a/b*, *b/a*, *a/L*, *b/L*) and visual results, the ratio of *a/b* and *b/a* were found to have the highest correlation coefficient with visual results (–0.934 and 0.941, respectively) [29]. In previous studies, the ratio *a/L* has been used to represent GC [8, 25]. Therefore, in this study, the ratios *a/b*, *a/L*, and visual results were used as GC indicators and designated as GC.a.b, GC.a.L, and GC.visual, respectively.

### Statistical analysis

Basic descriptive statistical analysis and analysis of variance (ANOVA) for the phenotypic data were carried out using R4.3.2 running in RStudio 2023.12.0 [35, 36]. The best linear unbiased prediction (BLUP) for PHS trait across multiple environments, broad-sense heritability (*H*^*2*^) and linear regression analysis were calculated by using the R package lme4. Pearson’s correlation coefficients among PHS and GC traits between different environments were constructed using the R package PerformanceAnalytics (https://github.com/braverock/PerformanceAnalytics)*.*

### SNP genotyping and linkage disequilibrium analysis

Genomic DNA was extracted from the young leaves by CTAB method and genotyped by Illumina 90 K SNP array at North Dakota State University [37]. The SNPs that containing missing rate ≤ 10% and minor allele frequency (MAF) ≥ 5% were retained with parameters --maf 0.05 --geno 0.1 --mind 0.2 by using Plink v1.90b7.2 [38]. Additionally, SNPs with unknown or multiple physical positions were discarded in this study. Finally, 13,606 polymorphic SNPs were retained and used for subsequent analysis.

The population structure of the tested population was previously assessed [30]. Linkage disequilibrium (LD), defined as the squared correlation coefficient (r²) of alleles between intra-chromosomal markers, was calculated using PLINK software. The LD decay distance across the whole genome and each sub-genome was then plotted using R. The size of LD blocks was estimated by keeping the r^2^ threshold at half of the LD decay value.

### GWAS and favorable allele identification

A GWAS was performed with GAPIT in R by using four models: MLM, MLMM, FarmCPU, and BLINK [39–43]. Additionally, the MLM model in Tassel was also utilized for association analysis [44]. Markers were identified as significantly associated with the traits after Bonferroni correction (0.05/n, where n = number of markers). Therefore, *P* < 3.67e–6, –log_10_(*P*) > 5.44 was used as the threshold. Haploview 4.2 software was used to check LD block between adjacent markers for PHS and GC [45]. Adjacent significant markers located in the region of LD decay were regarded as part of the same QTL. In this study, all physical positions of SNPs, QTLs and genes used their positions of IWGSC RefSeq v1.1. For convenience, the physical positions of SNPs within the IWGSC RefSeq v2.1 reference genome have also been provided. The physical locations of QTLs identified in this study were compared with those published QTLs and genes related to PHS, dormancy or color by using WheatOmics 1.0 (http://202.194.139.32/) and GrainGenes (https://wheat.pw.usda.gov/cgi-bin/GG3/browse.cgi) [46, 47].

Different alleles of each marker were used to determine the pyramiding effect of PHS resistant loci. Regression analysis was conducted between GP and the number of favorable alleles per cultivar. Alleles leading to lower GP were regarded as favorable alleles, whereas those leading to higher GP were considered unfavorable alleles.

### Putative gene prediction and expression analysis in silicon

Using the physical positions provided by IWGSC RefSeq v2.1, all genes located within the LD decay distance of associated SNPs were extracted from WheatOmics 1.0 (http://202.194.139.32/tools/intervalTools.html). Meanwhile, their corresponding homologous genes in *Arabidopsis* and rice were also acquired in WheatOmics 1.0 by using the gene IDs from IWGSC RefSeq v2.1 (http://202.194.139.32/homologtools/index.html). Putative candidate genes were prioritized based on the following criteria: (i) Genomic localization within regions harboring significant SNPs associated with PHS tolerance; (ii) Functional relevance to PHS tolerance, seed dormancy, or phytohormones; (iii) Annotation as transcription factors containing AP2, bZIP, or B3 DNA-binding domains or classified within the MYB superfamily; (iv) Annotation as chromatin remodeling factors; (v) Orthology to experimentally validated PHS/dormancy regulators in *Arabidopsis* or rice.

The expression data for these candidate genes, spanning root, stem, leaf, spike, grain, embryo proper, endosperm, and aleurone layer, were retrieved from Wheat Expression Browser (http://www.wheat-expression.com/) [48]. Additionally, the expression profile of four PHS-related genes (*TaVp*, *Tamyb10*, *TaPHS1*, and *TaQsd1*) were downloaded from the same database [9, 49–51]. Genes with detectable expression in grain tissues were selected, and their expression patterns were visualized by using TBtools software (v2.109) [52]. Genes that exhibit similar expression patterns with these PHS-related genes are considered the most promising candidate genes for further validation.

### KASP marker development and validation

The markers linked to six major QTLs for PHS resistance were converted into Kompetitive Allele-Specific PCR (KASP) markers (Table S2). Primers for these KASP markers were designed using the online tool at http://202.194.139.32/snprimer/. The association between these KASP markers and PHS resistance was validated using a natural population of 220 wheat varieties (Table S3), sourced from 23 countries (154 from China, including 79 cultivars and the rest landraces). The population was cultivated at the Ezhou Base of the Hubei Academy of Agricultural Sciences (114.66156°E, 30.39064°N) and harvested during 2023–2024. Genotyping of the KASP markers was performed by Wuhan Jingtai Biotechnology Co., Ltd. PHS resistance was assessed via spike-wetting tests (five spikes per variety), using the average germination percentage (GP) as an indicator. A Student’s t-test (R programming language) was conducted to evaluate allele effects on PHS resistance.

## Results

### Phenotypic variation and correlation between PHS and GC

A five-year spike-wetting test was conducted on a natural population to evaluate PHS resistance, with GP serving as the primary indicator (Table S1). Lower GP signified higher PHS resistance. Statistical analysis revealed a wide variation in GP within the population, ranging from 0.2 to 99.7% across various environments (Table 1). The coefficient of variation (CV) for GP was substantial, varying from 18.92% (GP16) to 46.84% (GR19), underscoring the high dispersion in PHS resistance within this population.

**Table 1:** Statistical analysis of PHS resistance and generalized heritability in 235 wheat cultivars

Trait	Min (%)	Max (%)	Mean (%)	SD (%)	CV(%)	H^2^(%)
GP15	11.6	95.3	62.3	18.66	29.93	85.34
GP16	30.4	99.2	76.6	14.50	18.92	
GP17	9.2	93	58.4	17.48	29.92	
GP18	1.2	99.7	62.7	24.73	39.42	
GP19	0.2	99.6	66.2	31.01	46.84	
BLUP	27.3	89.2	65.3	14.46	22.15	

**Fig. 1:**
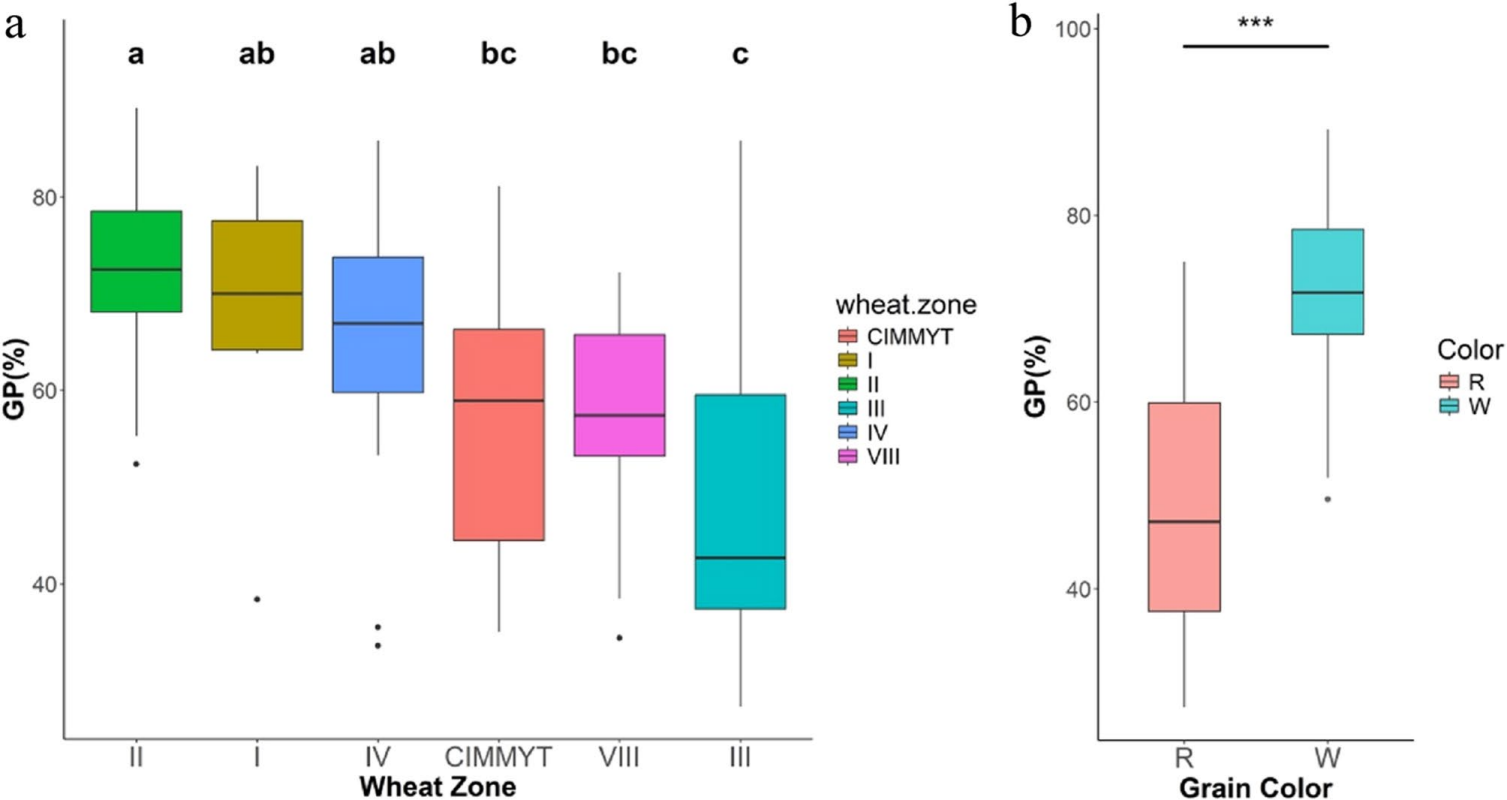
Boxplots for PHS resistance in BLUP environment grouped by wheat zone **a** and grain color **b**. GP (%) represents the germination percentage of spikes in spike-wetting tests. I: Northern Winter Wheat Zone; II: Yellow and Huai River Valleys Facultative Wheat Zone; III: Middle and Lower Yangtze Valleys Autumn-Sown Spring Wheat Zone; IV: Southwestern Autumn-Sown Spring Wheat Zone; VIII: Northwestern Spring Wheat Zone; CIMMYT: CIMMYT Wheat Zone; R: red-grained cultivars; W: white-grained cultivars. Wheat Zones with different letters indicate significant differences (*P* < 0.05) in GP, and those with the same letters show no significant differences (*P* > 0.05)

Six wheat zones were divided based on the origins of 235 cultivars (Table S1). The means of GP and BLUP values were employed to compare PHS tolerance among different zones (Fig. 1a; Table S4). Cultivars from Zone III, the Middle and Lower Yangtze River Valleys Autumn-Sown Spring Wheat Zone, exhibited the highest PHS resistance, as reflected by the lowest GP. This zone usually experiences rainfall during wheat harvest period and predominantly comprises red-grained wheat, which is known for enhanced PHS tolerance, as observed in this study (Fig. 1b). In contrast, cultivars from Zone II, the Yellow and Huai River Valleys Facultative Wheat Zone, which are exclusively white-grained, showed the lowest PHS tolerance.

**Fig. 2:**
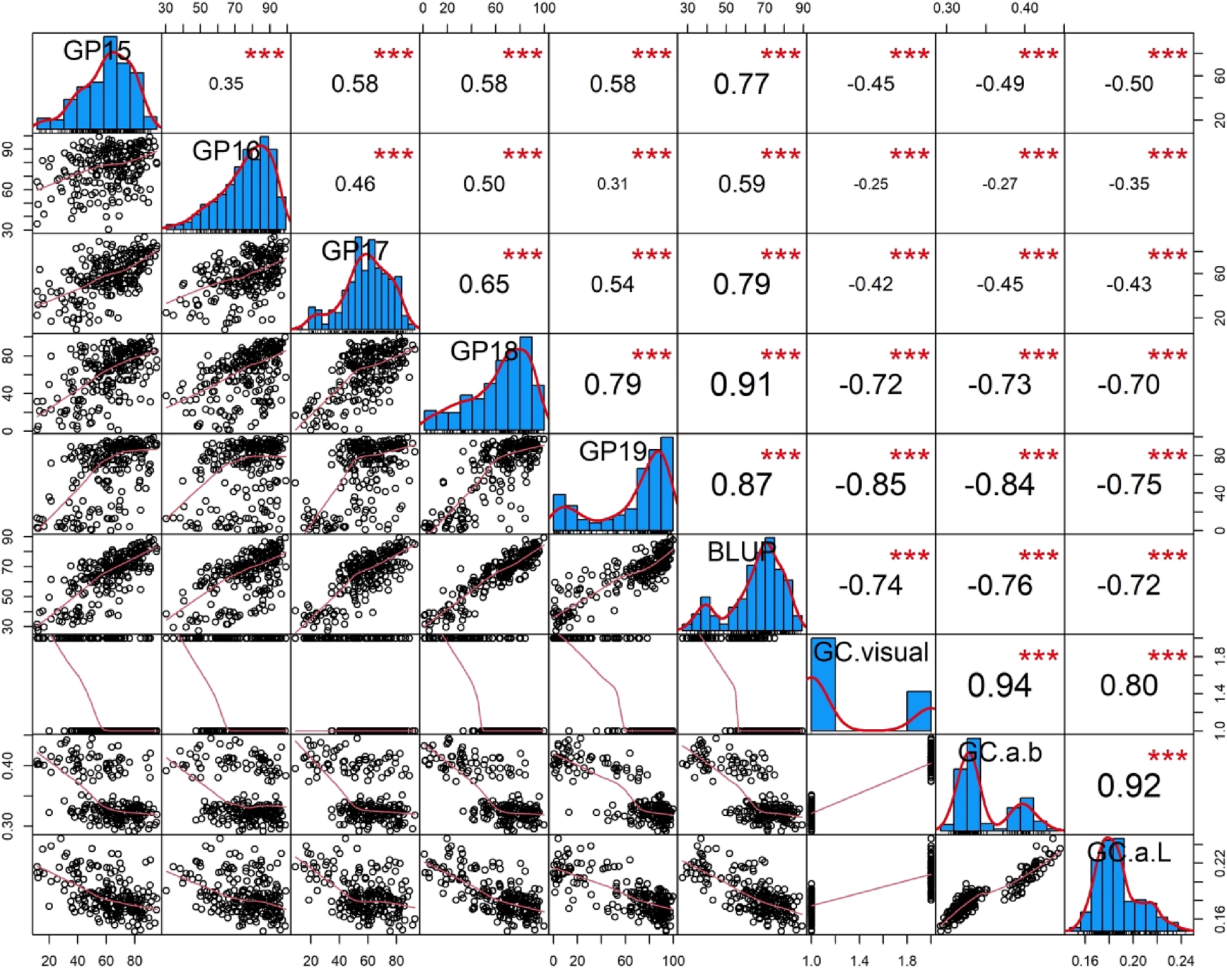
Correlation coefficients between PHS resistance and GC in 235 wheat cultivars. The notations of GP15, GP16, GP17, GP18, and GP19 represent the PHS resistance detected during the 2015–2019 growing seasons, specifically measured by germination percentage (GP). BLUP represents the best linear unbiased prediction (BLUP) for PHS resistance across multiple environments. GC denotes grain color, with GC.visual representing visually assessed traits and GC.a.b/GC.a.L representing colorimeter-derived ratios of a/b and a/L, respectively. *** indicates a significance level of *P* < 0.001 in Pearson’s correlation analysis

ANOVA showed that PHS trait was significantly affected by genotype, environment and genotype-by-environment interaction (*P* < 2e–16) (Table 2). The broad-sense heritability (*H*^*2*^) of PHS trait across five environments was 85.34% (Table 1), suggesting a robust and stable inheritance pattern, thereby making it amenable to genetic dissection. A significant positive correlation was observed for PHS trait across all environments, with correlation coefficients ranging from 0.31 to 0.91 (*P* < 0.0001) (Fig. 2).

**Table 2:** Variance analysis of PHS resistance in a natural population composed of 235 wheat cultivars

Variance	Sum of square	Mean square	F-value	*P* value
G	682,076	2915	31.538	< 2e-16
E	88,704	22,176	239.941	< 2e-16
G * E	435,723	468	5.064	< 2e-16

The GC traits (GC.visual, GC.a.b, GC.a.L) of the population were assessed through visual observation and a colorimeter (Table S1). Analysis of GC.a.b revealed distinct value ranges between wheat varieties with white grains (0.29–0.35) and those with red grains (0.37–0.44) (Table S5). Significant positive correlations were observed among the GC traits (Fig. 2). Additionally, a notable negative correlation was detected between the PHS trait and GC traits, further reinforcing the notion that red-grained wheat varieties exhibit enhanced tolerance to PHS. Notably, the GC.a.b trait displayed a superior ability to represent GC characteristics and exhibited a higher correlation coefficient of 0.84 with the PHS trait compared to the GC.a.L trait.

### Genome-wide association studies on PHS and GC

**Fig. 3:**
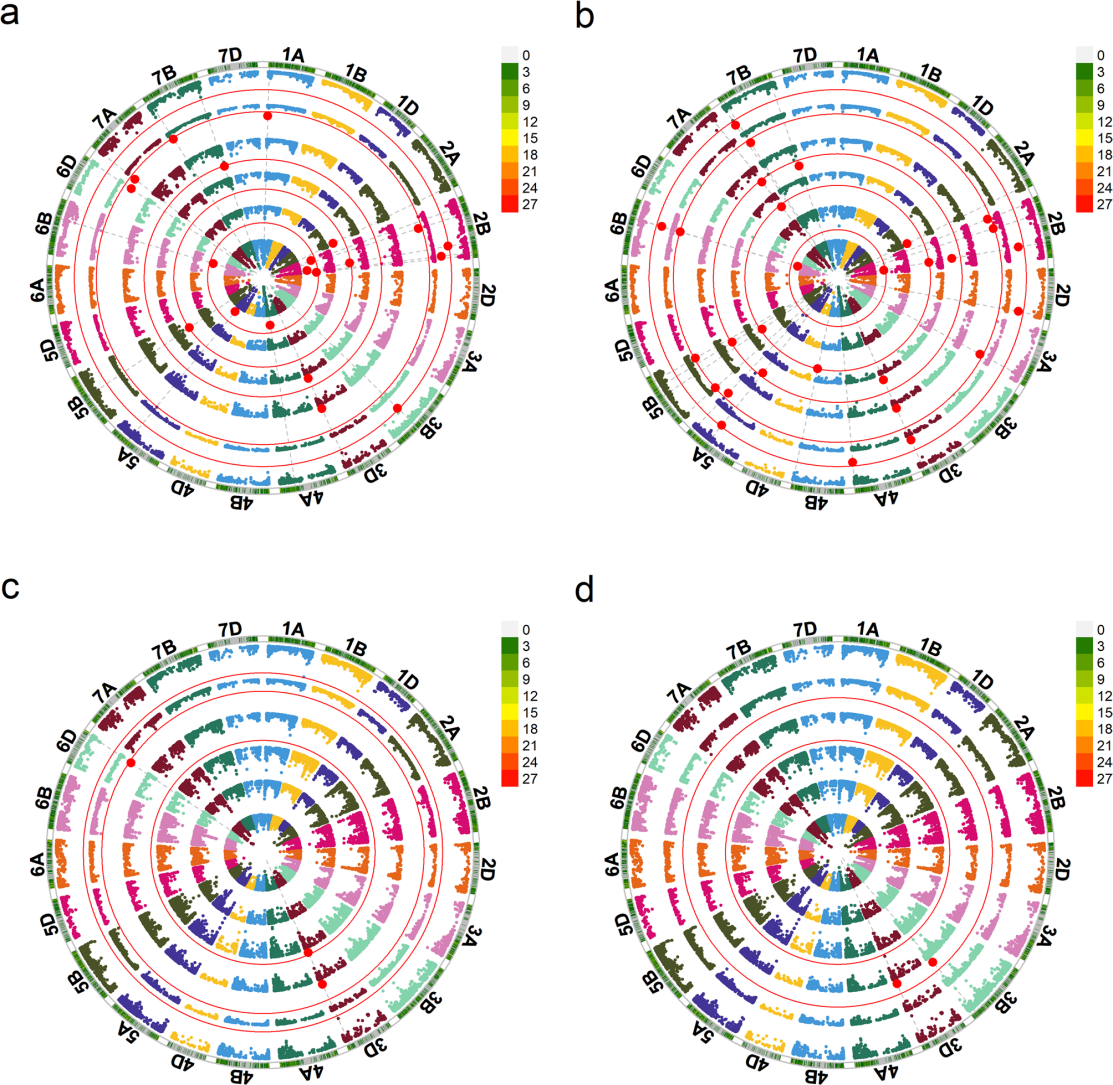
Manhattan plots for PHS trait by using Blink **a**, FarmCPU **b**, MLMM **c**, MLM **d** models in GAPIT. From the inner circle to the outer circle, these circles represent GP15, GP16, GP17, GP18, GP19, and BLUP environments. Red dots represent the significant markers

BLINK, FarmCPU, MLMM, and MLM models in GAPIT, along with MLM in Tassel, were employed for association analysis. The Manhattan and Q-Q plots generated by these models were drawn in R (Fig. 3, S2, S3). A total of 33 significant SNPs (*P* < 3.67e–6) were associated with PHS. LD analysis revealed varied LD decay distances across sub-genomes: 1.86, 2.57, 1.63, and 1.88 Mb for the A, B, D, and the whole genome, respectively (Fig. S4).

Ultimately, 30 QTLs were identified (Table S6), with a majority residing on the B sub-genome, in contrast to just three on the D sub-genome. Chromosomes 2B and 5B harbored six and four QTLs, respectively. Notably, 12 QTLs exhibited stability across more than two models or environments, particularly *QPhs.hbaas-2B.4*, *QPhs.hbaas-3D*, and *QPhs.hbaas-6B*. *QPhs.hbaas-2B.4*, linked to two SNPs, associated with PHS in four environments (Table 3). *QPhs.hbaas-3D*, detected by all five models in three environments, had the greatest impact, accounting for up to 49.7% of the phenotypic variation. *QPhs.hbaas-6B*, also comprising two SNPs, was found in three environments. Additionally, *QPhs.hbaas-2B.3*, *QPhs.hbaas-3B.2*, and *QPhs.hbaas-7B.3* were stable QTLs, explaining 10.0–35.5% of the phenotypic variation. The remaining QTLs had minor effects, explaining less than 10.0% of the phenotypic variation.

**Table 3:** 12 stable QTLs related with PHS and 4 common QTLs related with PHS and GC were found by association analysis

QTL	Marker	Chr.	v1.1 Pos(Mb)	v2.1 Pos(Mb)	Environment(Model)^a^	− log10 (*P*)	PVE	Reference
Qphs.hbaas-2 A*	Excalibur_rep_c104620_183	2 A	740.4	744.4	GP17(1,2)	9.1–12.1	0.1–6.1	
***Qphs.hbaas-2B.2***	wsnp_Ex_c269_518324	2B	118.3	126.2	GP16(1)	6.9	7.0	
***Qgc.hbaas-2B.2***	wsnp_Ku_c4042_7375053	2B	118.7	126.6	GC.visual(1)	6.8	4.7	
***Qphs.hbaas-2B.3***	Excalibur_c19344_137	2B	227.1	235.0	GP19(1,2)	6.1–8.5	2.3–10.0	
Qgc.hbaas-2B.3*	Excalibur_c19344_137	2B	227.1	235.0	GC.visual(1), GC.a.b(1,2)	6.6–10.2	1.7–7.4	
Qphs.hbaas-2B.4*	IACX5850/RAC875_c14775_1048	2B	599.8-600.7	607.6-608.5	GP16(1,2), GP17(1,2), GP18(2), BLUP(1,2)	5.5–8.9	0.0–8.0	
*Qphs.hbaas-3B.2*	GENE-1785_626	3B	760.1	775.5	GP19(4,5)	7.8–8.3	14.2–27.9	[50, 53, 54]
Qgc.hbaas-3B.3*	GENE-1785_626	3B	760.1	775.5	GC.visual(1,2,3,4,5), GC.a.b(1,2,3,4,5), GC.a.L(1,2)	7.4–37.6	0.0-66.9	[50, 55]
Qphs.hbaas-3D*	D_GA8KES402JVT1Y_74	3D	571.6	572.9	GP18(1,2,3), GP19(1,2,3,4,5), BLUP(2)	5.8–25.6	0.6–49.7	[2, 4, 8, 50, 53, 54, 56]
*Qgc.hbaas-3D*	D_GA8KES402JVT1Y_74	3D	571.6	572.9	GC.visual(4,5), GC.a.b(4,5),	6.3–10.2	0.0-18.1	[50, 55, 56]
Qphs.hbaas-5 A.1*	wsnp_Ex_c12440_19836844	5 A	417.9	418.1	GP18(2), BLUP(2)	5.8–6.8	7.2–7.8	
Qphs.hbaas-5B.2*	BobWhite_c8048_663	5B	403.2	406.2	GP17(1,2)	5.6–6.2	0.0-9.5	[54]
Qphs.hbaas-6B*	BS00106588_51/BS00022192_51	6B	470.9-472.6	479.0-480.8	GP16(1,2), GP19(2), BLUP(2)	5.7–7.1	0.0-7.9	[54]
Qphs.hbaas-6D*	BS00049818_51	6D	451.0	472.5	GP19(1,3)	6.3–12.0	3.1-7.0	
Qphs.hbaas-7 A.2*	IACX2471/JD_c149_1700	7 A	669.7-670.8	673.7-675.4	GP17(2), GP18(2)	6.2–6.4	2.4–5.8	
Qphs.hbaas-7B.2*	Tdurum_contig11028_236	7B	52.6	55.0	GP19(1,2)	6.5–7.7	0.0–4.0	[53, 54, 57]
Qphs.hbaas-7B.3*	Excalibur_c7338_563	7B	700.8	710.5	GP18(1,2)	5.6–7.6	0.0-35.5	[53, 54]

*12 Stable QTLs identified by more than two models or environments

^a^Numbers 1 to 4 represent the models BLINK, FarmCPU, MLMM, and MLM (implemented in GAPIT), respectively. Number 5 represents the MLM model used in Tassel

QTLs in bold are novel QTLs

A total of 27 QTLs were found to be significantly associated with GC (Table S7, Fig. S5). Nearly half of these QTLs were on B sub-genome. Among these, 12 were stable, detected across more than two models or GC traits. Six QTLs, *Qgc.hbaas-1 A.2*,* Qgc.hbaas-3 A.2*, *Qgc.hbaas-3B.3*, *Qgc.hbaas-3D*, *Qgc.hbaas-5B.2*, and *Qgc.hbaas-7B.1*, exhibited substantial effects on GC, accounting for 10.2–66.9% of the phenotypic variation. Notably, *Qgc.hbaas-3B.3*, detected by all five models in three GC traits, had the most significant impact on GC.

**Fig. 4:**
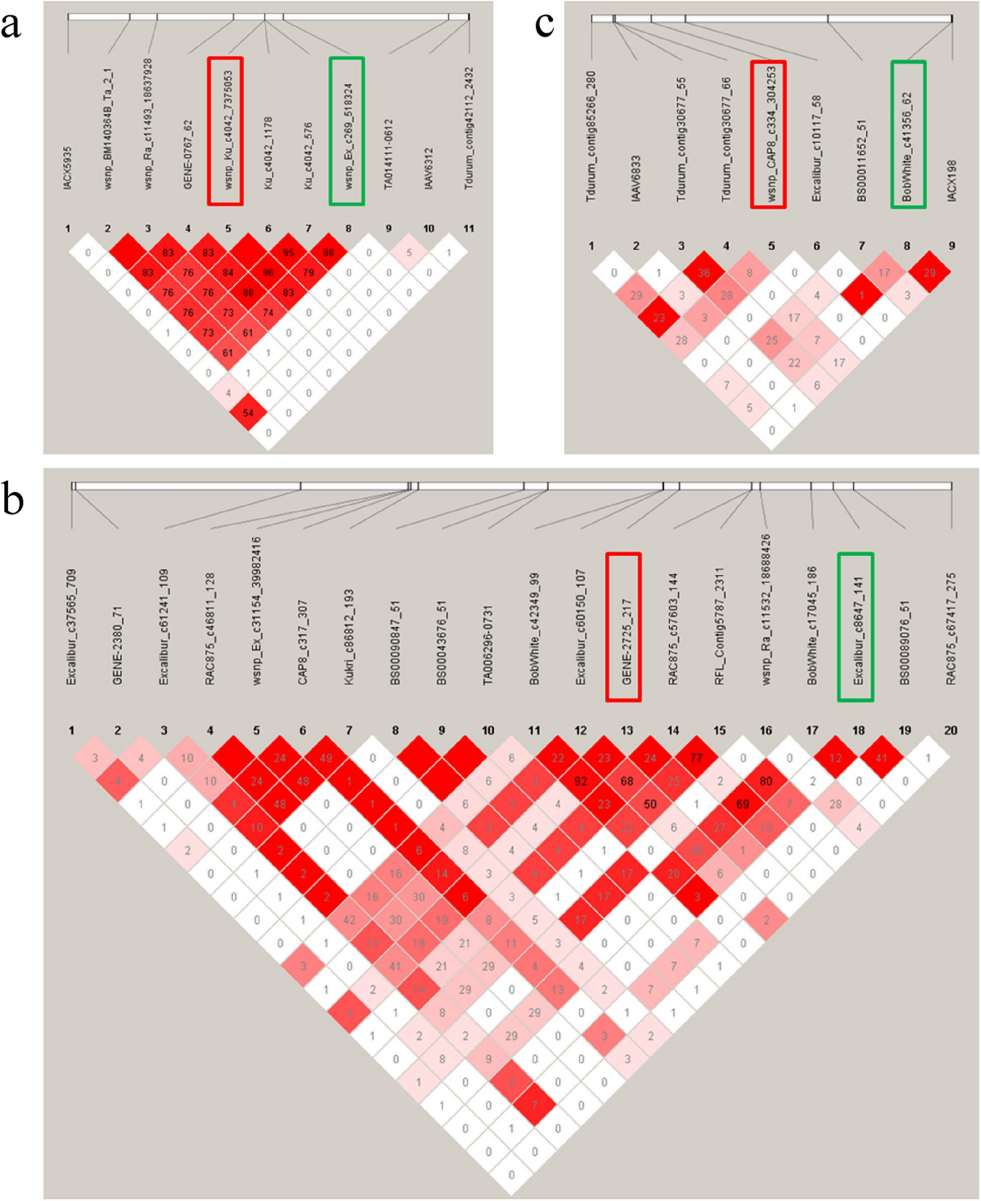
Linkage Disequilibrium (LD) analysis of markers on chromosomes 2B (113 Mb–123 Mb) **a**, 5 A (660 Mb–670 Mb) **b**, and 7B (6 Mb–10 Mb) **c**. The frames in green and red highlight significant markers that are associated with PHS resistance and GC, respectively. The number in square means the pairwise r^2^ value (displayed as percentages)

LD block analysis was conducted in HaploView to confirm the linkage of adjacent markers to either PHS or GC (Fig. 4). Notably, *Qphs.hbaas-2B.2* (*wsnp_Ex_c269_518324*, 118.7 Mb) and *Qgc.hbaas-2B.2* (*wsnp_Ku_c4042_7375053*, 118.3 Mb) were tightly linked (pairwise r^2^ = 0.836), suggesting that they reside within the same QTL. This may hint at the presence of a pleiotropic gene regulating both traits. Conversely, *Qphs.hbaas-5 A.3* (*Excalibur_c8647_141*, 666.9 Mb) and *Qgc.hbaas-5 A.2* (*GENE-2725_217*, 665.6 Mb), despite physical proximity, were located in distinct QTLs (r² = 0.018). Similarly, *Qphs.hbaas-7B.1* (*wsnp_CAP8_c334_304253*, 6.8 Mb) and *Qgc.hbaas-7B.1* (*BobWhite_c41356_62*, 9.8 Mb), separated by 3 Mb, also mapped to different QTLs (r² = 0.004). Ultimately, four common QTLs associated with both PHS and GC were detected on chromosomes 2B (2 loci), 3B (1 locus), and 3D (1 locus). *Qphs.hbaas-3B.2*/*Qgc.hbaas-3B.2* and *Qphs.hbaas-3D*/*Qgc.hbaas-3D* demonstrated significant impacts on both PHS and GC. Notably, the genes *Tamyb10-B1* (3B: 757,918,264–757,920,082) and *Tamyb10-D1* (3D: 570,801,163–570,803,376) are precisely located within the LD decay region of these two QTLs, implicating them as the causal genes regulating PHS resistance and GC. Additionally, *TaVP-3 A* (3 A: 659,552,136–659,556,535) was in close proximity to the LD decay region of *Qphs.hbaas-3 A* (LD decay region: 655,758,049–659,478,049), indicating that it might be the causal gene underlying this QTL.

### Pyramiding effect of favorable alleles

**Fig. 5:**
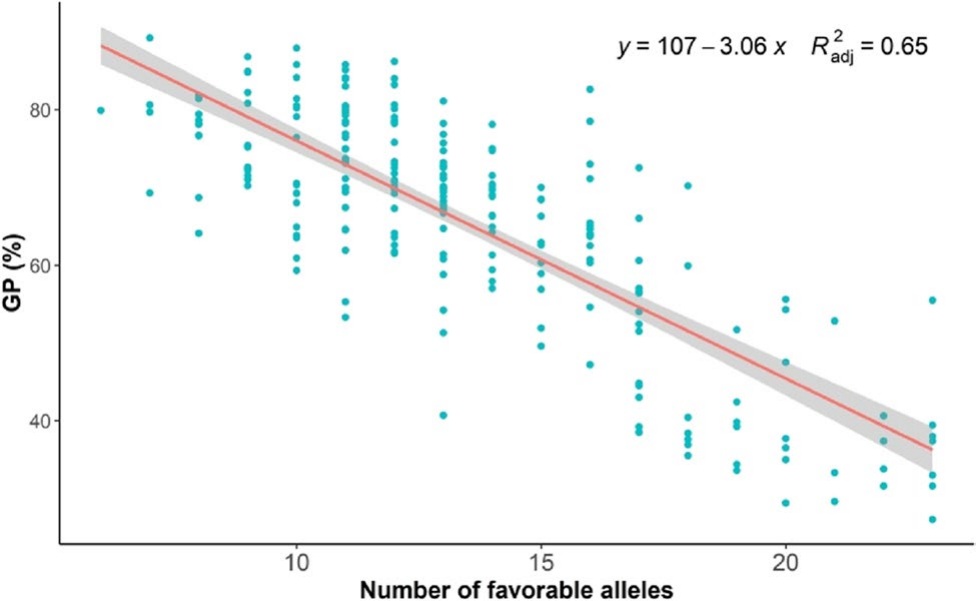
Linear regression between the number of favorable alleles and GP in BLUP environment

To assess the influence of favorable alleles on PHS resistance, the correlation between GP value and the number of favorable alleles across 235 cultivars was analyzed. The allele count ranged from 6 to 23, averaging 13.5 (Table S1). A notable inverse linear relationship (R^2^ = 0.65) was observed, suggesting a cumulative effect of favorable alleles in enhancing PHS resistance (Fig. 5). Cultivars rich in favorable alleles are invaluable for breeding PHS-tolerant wheat. Strategically pyramiding these alleles can enhance PHS resistance in new cultivars.

### Candidate gene identification of PHS

**Fig. 6:**
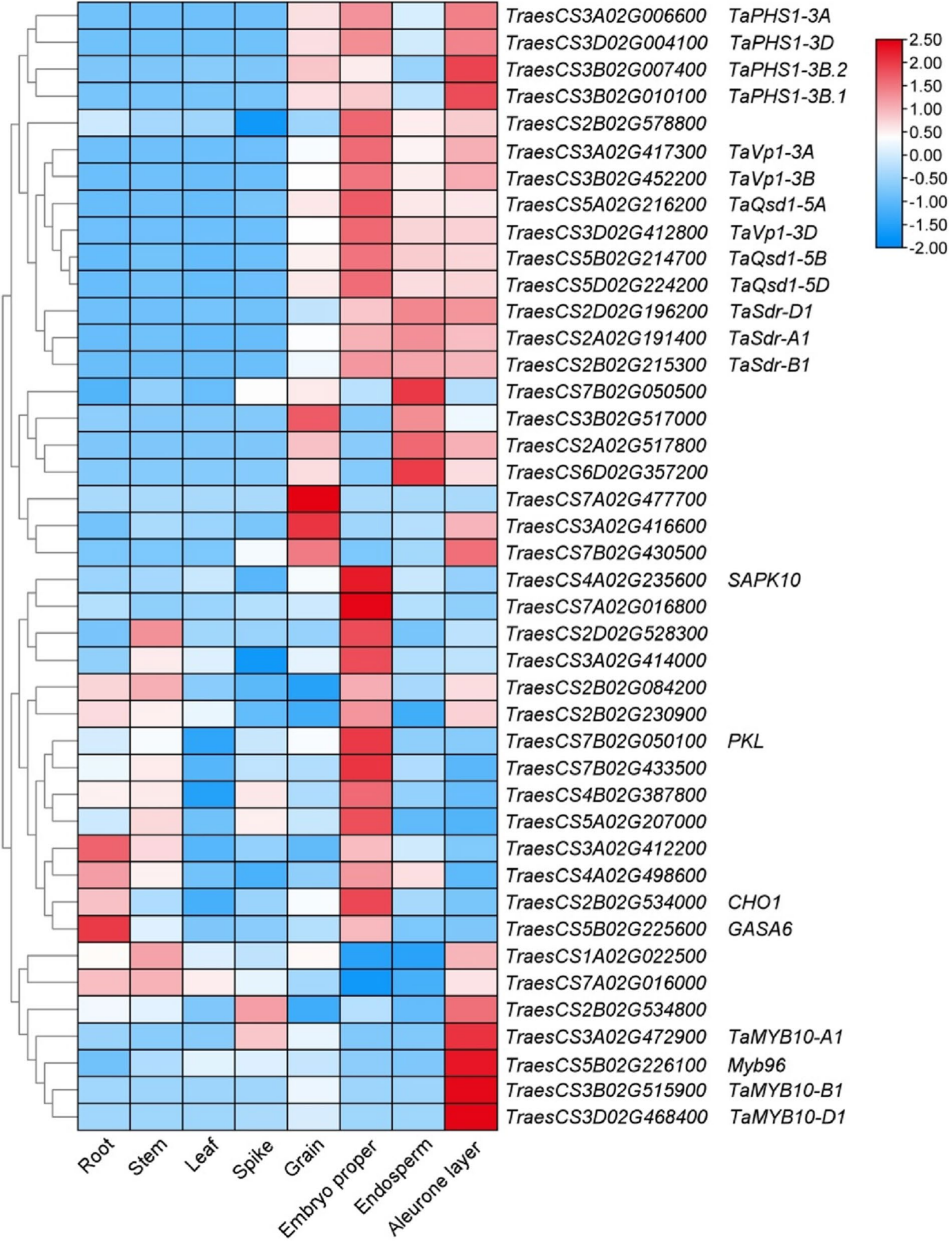
Heatmap represents the expression profile of genes and 26 candidate genes related to PHS resistance. The gene expression values are expressed as log2-transformed TPM (transcripts per million) values and have been normalized by row scaling

Based on the LD decay analysis, high-confidence genes within the 1.86-, 2.57-, 1.63-Mb flanking regions of associated SNPs in the A, B, and D sub-genomes were considered potential candidate genes, respectively. In total, 1636 high confidence (HC) genes were used for candidate gene prediction (Table S8). Upon thorough analysis of gene annotations, examination of protein domains, and assessment of homologs, a total of 90 genes were initially selected. Subsequently, 21 genes were eliminated due to their lack of expression in grain. The remaining 69 genes were then subjected to analysis of their expression patterns, alongside *TaPHS1*, *TaVp*, *TaQsd1*, *TaSdr*, and *Tamyb10*, utilizing TBtools (Fig. S6; Table S9). Apart from the candidate genes *Tamyb10-B1*, *Tamyb10-D1* and *TaVP-3 A* mentioned above, 26 genes were hypothesized to be associated with PHS resistance and thus require additional validation (Fig. 6; Table S10).

The genes *TaPHS1*, *TaVp*, *TaQsd1*, and *TaSdr* associated with PHS resistance, were clustered together and highly expressed in embryo, aleurone and endosperm. *TraesCS2B02G578800*, with similar expression pattern, was also grouped within this cluster. Additionally, seven genes predominantly expressed in seeds, endosperm, or aleurone, along with fourteen genes highly expressed in embryo, were also grouped alongside *TaPHS1*, *TaVp*, *TaQsd1* and *TaSdr*. *Tamyb10* alleles, highly expressed in aleurone, were grouped into a separate cluster. *TraesCS1A02G022500*, *TraesCS7A02G016000*, *TraesCS2B02G534800* and *TraesCS5B02G226100* were clustered with *Tamyb10* alleles due to their similar expression patterns. Notably, *TraesCS5B02G226100*, annotated as MYB transcription factor, shares homology with *Arabidopsis MYB96*, which is related to seed dormancy.

### Validation of KASP markers of the major QTLs for PHS resistance

**Fig. 7:**
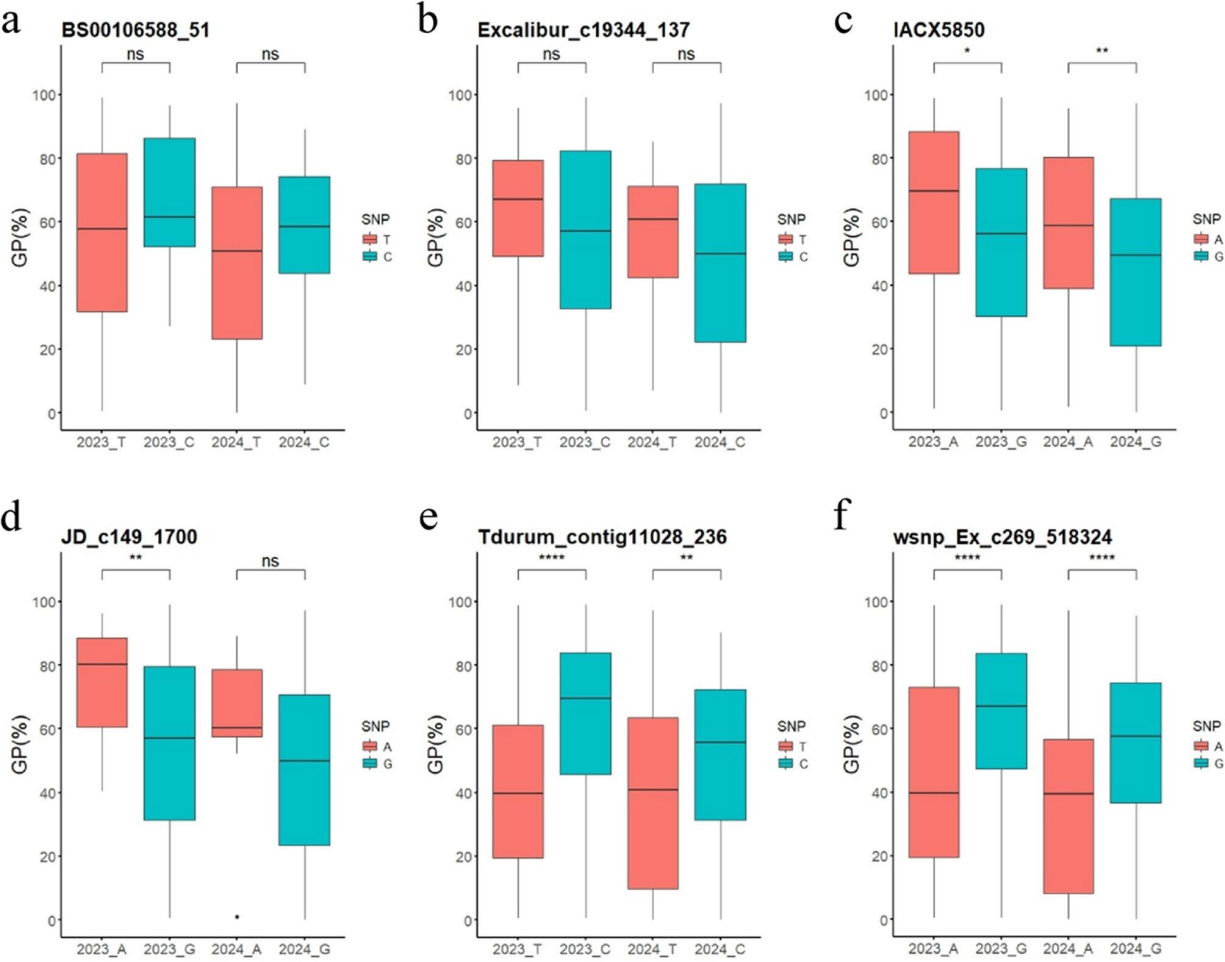
Box plots showing the distribution of GP values for six KASP markers in a natural population of 220 varieties, assessed over the years 2023 and 2024. (a-f). The six KASP markers were closely linked with QTL *Qphs.hbaas-6B*, *Qphs.hbaas-2B.3*, *Qphs.hbaas-2B.4*, *Qphs.hbaas-7 A.2*, *Qphs.hbaas-7B.2*, *Qphs.hbaas-2B.2*, respectively. Significance levels are indicated as follows: ∗∗∗∗ for *P* < 0.0001, ∗∗∗ for *P* < 0.001, ∗∗ for *P* < 0.01, ∗ for *P* < 0.05, and ns for non-significant differences

The association between six KASP markers and PHS resistance in a natural population of 220 wheat varieties across two environments was evaluated (Fig. 7; Table S11). Three KASP markers, *IACX5850*, *Tdurum_contig11028_236*, and *wsnp_Ex_c269_518324*, had significant effects on PHS resistance in two environments. For *IACX5850*, the favorable allele G (present in 73.2% of varieties) had a GP range of 44.55–53.08%, significantly lower than the 58.11–64.37% for allele A. For *Tdurum_contig11028_236* (39.44% favorable allele) and *wsnp_Ex_c269_518324* (38.53% favorable allele), varieties carrying the favorable alleles had GP ranges of 40.24–44.41% and 38.43–45.17%, respectively, both significantly lower than the GP ranges of 52.50–62.60% and 54.43–62.91% for unfavorable alleles. Marker *JD_c149_1700* significantly affected PHS resistance only in the 2023 environment. Though its favorable allele G also showed a lower GP than allele A in 2024, this was not statistically significant. Markers *BS00106588_51* and *Excalibur_c19344_137* had no significant effect on PHS resistance, yet varieties with their favorable alleles showed lower GP in both environments. Therefore, KASP markers *IACX5850*, *Tdurum_contig11028_236*, and *wsnp_Ex_c269_518324* are promising for wheat breeding to improve PHS resistance.

## Discussion

### Employing diverse models in association mapping enhances QTL discovery

Tassel and GAPIT software are two prominent tools widely utilized in association analysis. MLM is a single-locus test often used in GWAS, but can yield high false negatives due to its stringent control of false positives. In this study, only two highly significant QTLs were identified by MLM, indicative of substantial false negatives. Some studies have adopted less stringent thresholds to mitigate this issue, albeit at the risk of introducing false positives. To overcome the limitations of MLM, multi-locus GWAS methods such as MLMM, FarmCPU and BLINK have been developed and integrated in GAPIT. The percentage of PVE by associated markers in GAPIT is linked to their MAF and the magnitude of their marker effect [43]. Conversely, non-associated markers are considered to contribute negligibly to the total variance. In this study, some significant markers identified by GAPIT exhibited high PVEs, such as *Qgc.hbaas-3B.3* (PVE = 66.9%), potentially attributable to a combination of factors: the detection of fewer associated markers within the specific environment, the high MAF of these markers and the magnitude of their marker effect. Additionally, different PVEs were showed in Tassel and GAPIT by using same MLM model. So MLM model in Tassel was also implemented in this study to give a reference with other studies.

In our GWAS analysis, a stringent Bonferroni correction method was employed. Only two QTLs, *Qphs.hbaas-3B.2* and *Qphs.hbaas-3D* were identified to relate to PHS by using MLM in Tassel and GAPIT. These QTLs, harboring *Tamyb10-B1* and *Tamyb10-D1* respectively, had large effects on PHS resistance. While using BLINK and FarmCPU in GAPIT, 17 and 20 QTLs were detected, respectively. Notably, 9 and 10 of these QTLs have been previously reported, underscoring the reliability of our findings. However, *Qphs.hbaas-3B.2* was not found by either BLINK or FarmCPU, highlighting potential limitations of these models. A similar trend was observed in the association mapping of GC, where only *Qgc.hbaas-3B.3* and *Qgc.hbaas-3D*, harboring *Tamyb10-B1* and *Tamyb10-D1*, were identified by using MLM. In contrast, 16 and 20 QTLs were uncovered using BLINK and FarmCPU, respectively. *Qgc.hbaas-3D* was not detected by these two methods. Additionally, only two and three QTLs related to PHS and GC were identified by MLMM. *Qphs.hbaas-3B.2* and *Qgc.hbaas-3D* were also absent when using MLMM. These observations suggest that under the rigorous Bonferroni correction framework, employing multiple statistical models is crucial for enhancing QTL discovery and mitigating the risk of overlooking significant associations.

### Visual inspection and a/b value: superior for identifying PHS-associated color loci

Previous studies have established a correlation between PHS resistance and GC, with red-grained wheat often exhibiting greater resistance to PHS than white-grained varieties [4, 26]. Using the NaOH soaking method to evaluate GC, *Tamyb10-A1* and *Tamyb10-D1* loci were detected as being associated with GC and PHS resistance [55, 56]. Colorimeter parameters (L, b, Chroma and WI) were significantly related to the GP in wheat, with correlation coefficients ranging from 0.24 to 0.38 [58]. The *Tamyb10-B1* locus was identified through association mapping of PHS resistance and GC, utilizing the a/L parameter as an indicator of GC [25].

Our preliminary research found that Colorimeter parameters (L, a, b, a/b, b/a, a/L and b/L) were significantly correlated with PHS resistance, with a/b and b/a exhibiting the highest correlation coefficients [29]. In the current study, GC was evaluated through both visual inspection and quantitative assessment using the a/b and a/L values. Our results showed stronger correlations between PHS and visual inspection (*r* = 0.85) and the a/b ratio (*r* = 0.84), compared to a/L (*r* = 0.75) (Fig. S7). Furthermore, 16 and 15 QTLs were detected in QTL mapping using visual inspection and the a/b value, respectively, both of which harboring *Tamyb10-B1* and *Tamyb10-D1* genes. In contrast, only 10 QTLs were identified by using the a/L value, with the *Tamyb10-D1* locus remaining undetected. Regarding the co-localization of QTLs for PHS and GC, 4, 3 and 1 common QTLs were identified, respectively, when visual inspection, the a/b and a/L value were used to evaluate GC. Consequently, this study confirmed that the utilization of visual inspection and the a/b value is more effective in identifying color loci associated with PHS traits compared to the a/L value.

### Common QTLs related to PHS resistance and GC

*Tamyb10* is the causal gene for the GC *R-1* locus and has been identified as responsible for PHS resistance and GC in many studies [25, 26, 55, 56]. The function of *Tamyb10-D1* in regulating PHS and GC has been established. Firstly, it can bind to the promoter of *NCED* (a gene related to ABA synthesis), thereby promoting *NCED* transcription, which in turn increases ABA content in the seed and inhibits seed germination. Secondly, it potentiates the expression of genes involved in the flavonoid biosynthetic pathway, encompassing CHS, CHI, F3H, GT and DFR, resulting in a darker GC phenotype [13]. Additionally, restoring the function of *Tamyb10-B1a* allele through CRISPR/Cas9-mediated editing can enhance seed dormancy and GC [59]. In our study, four common QTLs linked to PHS and GC were identified, with *Qphs.hbaas-3B.2*/*Qgc.hbaas-3B.2* (harboring *Tamyb10-B1*) and *Qphs.hbaas-3D*/*Qgc.hbaas-3D* (harboring *Tamyb10-D1*) being major ones. Additionally, two novel QTLs related to PHS resistance and GC were identified. Notably, *Qphs.hbaas-2B.3/Qgc.hbaas-2B.3* was stable and found to account for up to 10% and 7.4% of the phenotypic variation in PHS resistance and GC, respectively. Further investigation into the genes underlying the new QTLs will deepen our genetic understanding of the intricate interplay between PHS resistance and GC. The work will provide marker-assisted selection (MAS) strategies for the development of crop varieties with enhanced PHS resistance and desirable GC characteristics.

### Candidate genes related to PHS resistance or dormancy

In our study, *Tamyb10-B1*, *Tamyb10-D1*, *TaVp-1* and other 26 genes potentially relate to PHS resistance were selected through a comprehensive analysis including gene annotations, examination of protein domains, assessment of homologs and expression pattern analysis (Table S10). As previously mentioned, *Tamyb10-B1* and *Tamyb10-D1* should be the causal genes of *Qphs.hbaas-3B.2*/*Qgc.hbaas-3B.2* and *Qphs.hbaas-3D*/*Qgc.hbaas-3D*, respectively, in the regulation of PHS resistance and GC. *TaVp-1* is a homologous gene of the maize *Viviparous-1* (*Vp-1*) gene, which has been proven to be associated with seed dormancy [12]. Therefore, *TaVp1-3 A* might be the causal gene underlying *Qphs.hbaas-3 A*. However, this QTL was only detected in one environment and had minor effect on PHS resistance.

Among these candidate genes, six were identified as the most promising candidates due to their homology with genes known to regulate PHS or seed dormancy in other plant species (Table S12). *TraesCS2B02G578800* exhibited a similar expression pattern to *TaPHS1*, *TaVp*, *TaQsd1* and *TaSdr*. *TraesCS2B02G578800* is annotated as the Phosphoribosylformylglycinamidine synthase subunit PurQ with the ThiJ/PfpI domain. It shows homology to *OsDJ-1 C* and *AtDJ-1D*. Overexpression of *OsDJ-1 C* in rice has been found to improve roots, photosynthesis, yield and stress tolerance by detoxification of methylglyoxal (MG) [60]. Similarly, *AtDJ-1D*, through MG detoxification and deglycase activity, resulted in superior growth and tolerance to stress in transgenic plants [61]. MG, a toxic by-product of the glycolysis pathway in maturing or imbibed seeds, has been shown to inhibit seed germination [62]. GERMINATION-IMPAIRED GLYOXALASE 1 (*GIG1*), a glyoxalase I gene, has also been found to enhance seed germination by detoxifying MG [63]. Therefore, it is plausible that *TraesCS2B02G578800*, as a homolog of genes involved in MG detoxification, may potentially impact seed germination.

*TraesCS2B02G534000* might be the causal gene for *Qphs.hbaas-2B.5*, a novel QTL that was only detected in one environment. *TraesCS2B02G534000* is annotated as AP2-like ethylene-responsive transcription factor containing the AP2 domain. It is highly expressed in the embryo. It is homologous to *OsPLT1* (*LOC_Os04g55970*) in rice and shares similarity with AINTEGUMENTA-like 5 (*AIL5*/*CHO1*, *AT5G57390*) in *Arabidopsis*. *OsPLT1* has been confirmed to regulate root architecture [64]. *CHO1* was found to be mainly expressed in seed, especially in imbibed seeds. And it could enhance seed dormancy by acting downstream of ABA to repress GA biosynthesis [17, 65]. Thus, based on its expression pattern and the functional roles of its homologs, *TraesCS2B02G534000* is speculated to play a potential role in seed dormancy or germination.

*TaSnRK2.10–4 A* (*TraesCS4A02G235600*) is a member of the SnRK2 (sucrose non-fermenting 1-related protein kinase 2) family. It has been validated to be associated with thousand-grain weight (TGW), spike length, plant height and drought tolerance [66, 67]. Furthermore, heterologous overexpression of *TaSnRK2.10-4B* gene in rice resulted in a delayed germination phenotype and augmented sensitivity to exogenous ABA [67]. This suggests that the gene may influence seed dormancy and germination processes by regulating ABA signaling pathway. Besides, *TaSnRK2.10* shares high similarity with *SAPK8*, *SAPK9* and *SAPK10* in rice, as well as with *Snrk2.2/SRK2D*, *SnRK2.6/SRK2E/OST1*, *Snrk2.3/SRK2I* in *Arabidopsis*. *SAPK8*, *SAPK9* and *SAPK10* are members of the rice *SnRK2* family and are reported to be activated by ABA, participating in ABA signaling transduction [68]. ABA promotes jasmonic acid (JA) biosynthesis through the “SAPK10-bZIP72-*AOC*” pathway to inhibit seed germination in rice [69]. In *Arabidopsis*, *Snrk2.2*, *Snrk2.3* and *SnRK2.6* are functionally redundant ABA-activated SNF1-related protein kinases. Mutant lines harboring disruptions in two or three of these genes exhibited reduced seed dormancy [70, 71]. Therefore, it is speculated that *TaSnRK2.10–4 A* serves as a candidate gene related to seed dormancy.

Two genes, *TraesCS5B02G225600* and *TraesCS5B02G226100*, are candidate genes for *Qphs.hbaas-5B.2*. *TraesCS5B02G225600*, also known as *TaGASR24*, a gibberellin-regulated family protein, belongs to the GASA gene family and is regulated by ABA, GA and cold temperature. Moreover, another family member, *TaGASR34*, has been identified as associated with seed dormancy [72]. *TaGASR24* and *TaGASR34* share similarity to Gibberellic Acid-Stimulated Arabidopsis6 (*GASA6*, *AT1G74670*) and *GASA4* in *Arabidopsis*. *GASA6* was found to highly express in developing seeds and the embryonic axis of imbibed seeds. Overexpression of *GASA6* in seeds treated with glucose, ABA and PAC (a GA antagonist) resulted in significantly faster germination rates compared to wild-type seeds, whereas RNAi-mediated functional deficiency of *GASA6* led to notably slower germination rates. GASA6 is regulated by RGL2 (The DELLA protein REPRESSOR OF ga1-3-LIKE 2) and promotes the expression of *AtEXPA1*, thereby facilitating rapid seed germination [73]. Additionally, GASA4 has been shown to be induced by GA independently of light and is involved in seed development and germination [74].

*Tamyb31-B* (*TraesCS5B02G226100*) has been found to be related to benzoxazinoid biosynthesis and drought tolerance [75, 76]. Ectopic expression of *Tamyb31-B* in *Arabidopsis* conferred hypersensitivity to ABA, leading to impaired germination and reduced root growth in seedlings. *Tamyb31-B* exhibits a similar expression pattern with three *Tamyb10* homologs and shares considerable sequence homology with *MYB30*, *MYB31*, *MYB94*, and *MYB96* in *Arabidopsis*. Among these, *MYB30* and *MYB96* have been functionally implicated in seed dormancy regulation. *MYB30* was found to facilitate germination by promoting the expression of the ABA catabolic gene *CYP707A2* in response to nitric oxide (NO). Conversely, ABA represses the transcriptional activity of *MYB30* by ABA receptors such as PYR1/PYL/RCAR, leading to the suppression of *CYP707A2* and subsequently enhancing seed dormancy [77]. Furthermore, MYB96 actively promotes the expression of ABA biosynthetic genes (*NCED2*, *NCED5*, *NCED6*, *NCED9*) while inhibiting the expression of GA biosynthetic genes (*GA3ox1* and *GA20ox1*), thereby positively regulating seed dormancy [78]. Additionally, MYB96 binds to the promoter of *ABI4*, enhancing its expression. *ABI4*, in turn, binds to the promoters of ABA catabolic genes *CYP707A1* and *CYP707A2*, inhibiting their expression, and also promotes the expression of the GA metabolic gene GA2ox7, collectively contributing to the promotion of dormancy [78–82].

*Qphs.hbaas-7B.2*, a stable locus for PHS resistance, has been identified in this study and previously reported [53, 54, 57]. *TraesCS7B02G050100* is a candidate gene for this locus, and its homologs on 7 A and 7D chromosomes are also implicated in PHS resistance [54]. *TraesCS7B02G050100* encodes an ATP-dependent chromatin-remodeling factor CHD3, which is highly expressed in roots, spikes and seeds, with the highest expression levels observed in embryos. This gene is homologous to the *PICKLE* (*PKL*, *AT2G25170*) gene in *Arabidopsis*. *PKL* regulates gene transcription by modulating the H3K27me3 levels of target genes, playing a crucial role in various developmental processes and environmental responses, including embryogenesis, root meristem activity, photomorphogenesis, and thermomorphogenesis [3, 83]. On one hand, *PKL* inhibits the expression of key regulators of plant embryogenesis, such as LEAFY COTYLEDON1 (LEC1) and LEC2, thereby affecting the expression of ABA- and GA-related genes and ultimately inhibiting seed dormancy. On the other hand, recruited by the evening complex EARLY FLOWERING3 (ELF3)-ELF4-LUX ARRHYTHMO (LUX), PKL binds to the regulatory region of the positive regulator of seed dormancy, DOG1, inhibiting its expression and subsequently suppressing seed dormancy [24, 84]. Recent research has revealed that *TaPKL-7 A/7B/7D* (*TraesCS7A02G147100*,* TraesCS7B02G050100*,* TraesCS7D02G148900*) do not influence seed germination or PHS resistance [85]. However, RNA interference (RNAi)-mediated suppression of homologous *TaPKL* genes, namely *TaPKL-7 A/7B/7D*, *TaPKL-4 A/4B/4D*, and *TaPKL-3 A/3D*, has been shown to regulate PHS. Furthermore, *TaPKL-7 A/7B* have been implicated in modulating grain number per spike. Collectively, these findings underscore the necessity for further studies to elucidate the functional roles of *TaPKL* genes.

## Conclusions

Twelve stable loci related to PHS resistance were identified using the wheat 90 K SNP assay across 235 wheat varieties. *Qphs.hbaas-2 A*, *Qphs.hbaas-2B.3*, *Qphs.hbaas-2B.4*, *Qphs.hbaas-5 A.1*, *Qphs.hbaas-6D*, and *Qphs.hbaas-7 A.2* were probably novel. Additionally, our analysis unveiled two novel QTLs that exhibited a dual association with both PHS resistance and GC. Twenty-six potential candidate genes for PHS resistance were screened out. Among them, six genes stood out as highly promising candidates requiring further validation. Furthermore, three KASP markers, namely *IACX5850*, *Tdurum_contig11028_236*, and *wsnp_Ex_c269_518324*, which are linked with three major QTLs (*Qphs.hbaas-2B.2*, *Qphs.hbaas-2B.4*, and *Qphs.hbaas-7B.2*), can be used for marker-assisted selection for PHS resistance in wheat breeding. Notably, the a/b ratio derived from colorimeter measurements emerged as a valuable tool for precisely identifying these dual-associated QTLs, thereby facilitating the genetic dissection of their complex interactions.

## Supplementary Information

Supplementary Material 1.

Supplementary Material 2.

## Data Availability

The datasets used and/or analyzed during the current study are available to the corresponding author upon reasonable request.
