# Supplementary material for: Genome-wide association study of pre-harvest sprouting resistance and grain color in common wheat (Triticum aestivum L.)
Source: BMC Plant Biol. 2025 Jul 29;25:973. doi: 10.1186/s12870-025-07039-y (PMC12306042; doi:10.1186/s12870-025-07039-y)
Supplement: Supplementary file 1 — Supplementary Material 1. [file 12870_2025_7039_MOESM1_ESM.docx]

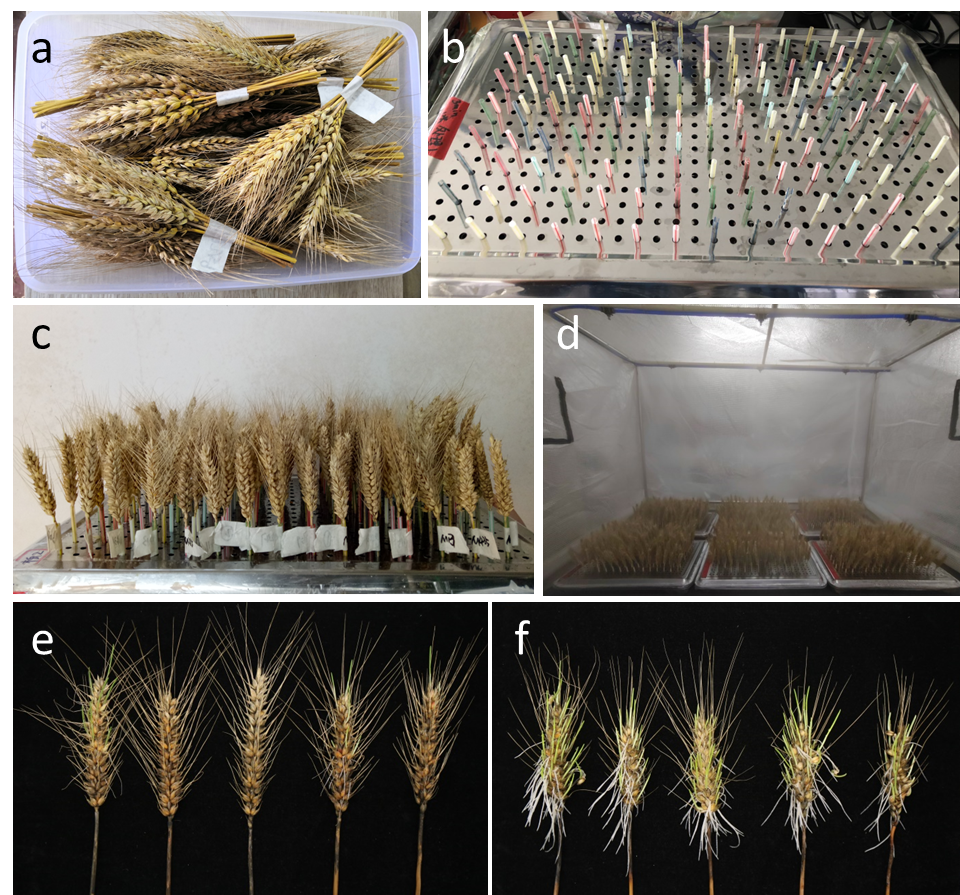


**Figure S1.** Spike-wetting test. (a) Spikes stored in plastic boxes to prevent breakage. (b) Trays with plastic straws used to maintain spike straight during testing. (c) Spikes thawed and kept at room temperature for 2 days before testing. (d) Simulated rainfall chamber with overhead sprayer. (e-f) Germination images of GWAS123 and GWAS172 cultivars after spike-wetting test.


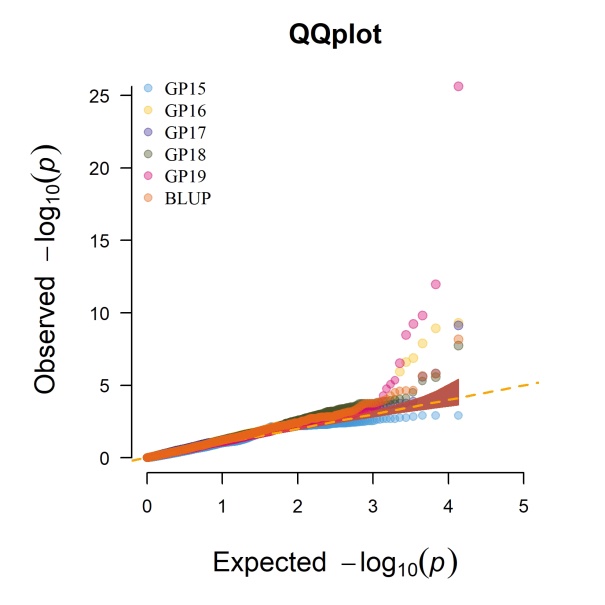

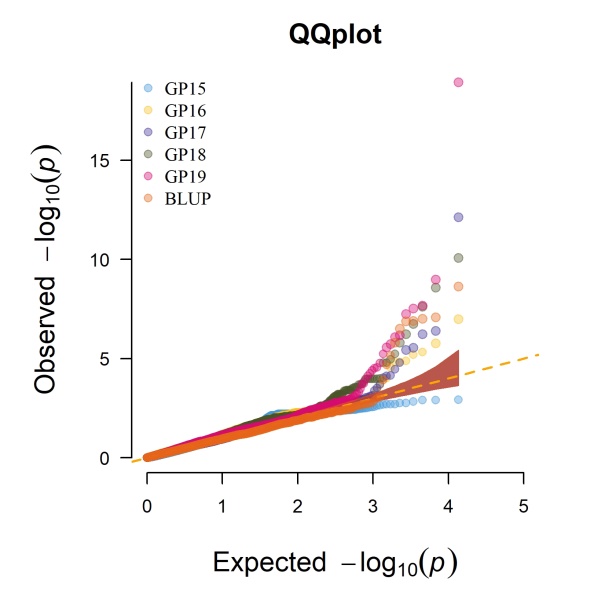

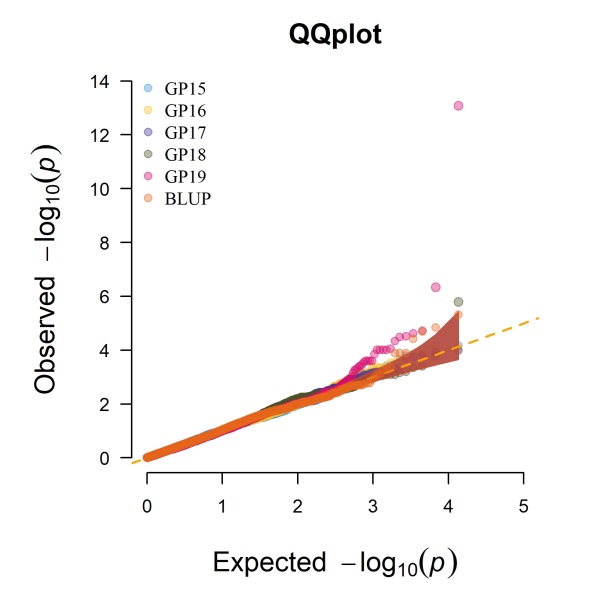

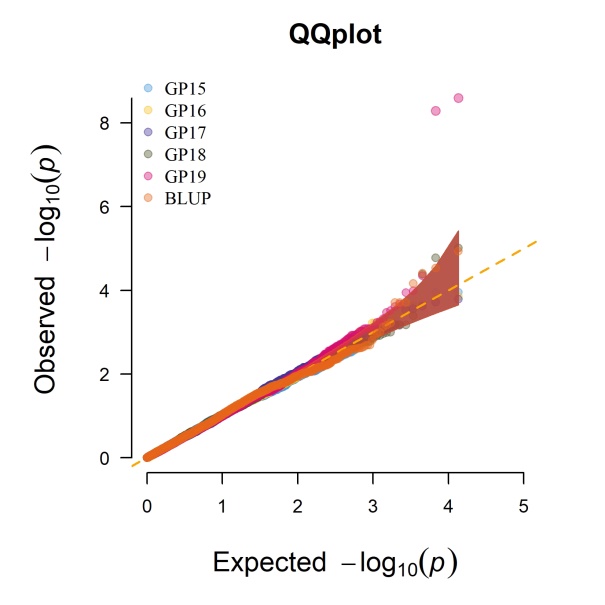


d

c

b

a

**Figure S2.** Q-Q plots for PHS trait by using Blink (a), FarmCPU (b), MLMM (c), MLM (d) models in GAPIT.


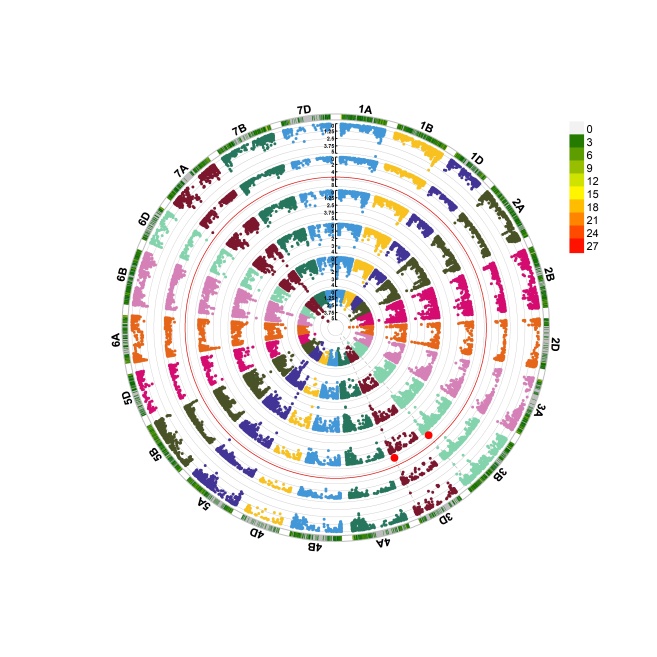

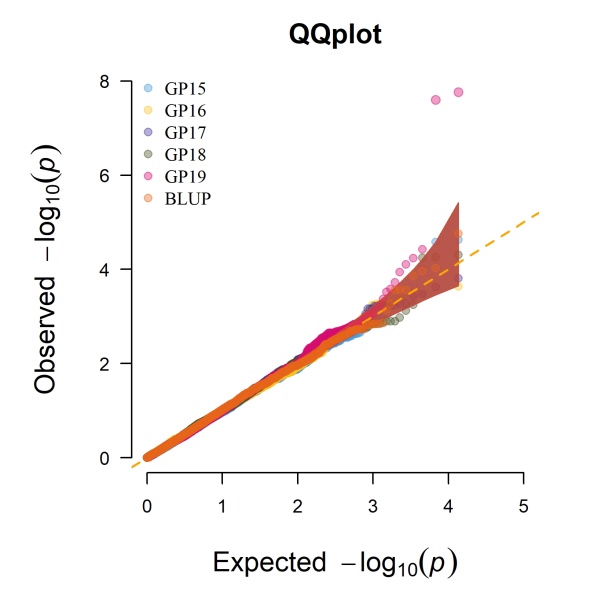


a

b

**Figure S3.** Manhattan plot and Q-Q plot for PHS trait by using MLM model in Tassel. Red dots in (a) and pink dots in (b) represent the significant markers.


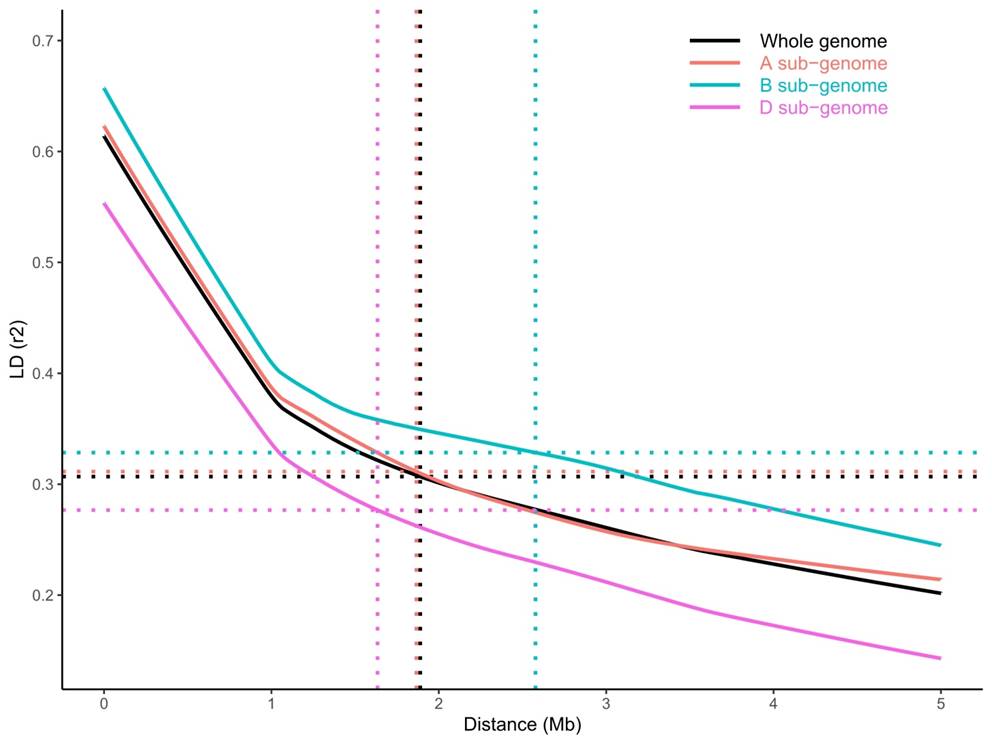


**Figure S4.** An LD decay plot was drawn using R.

**
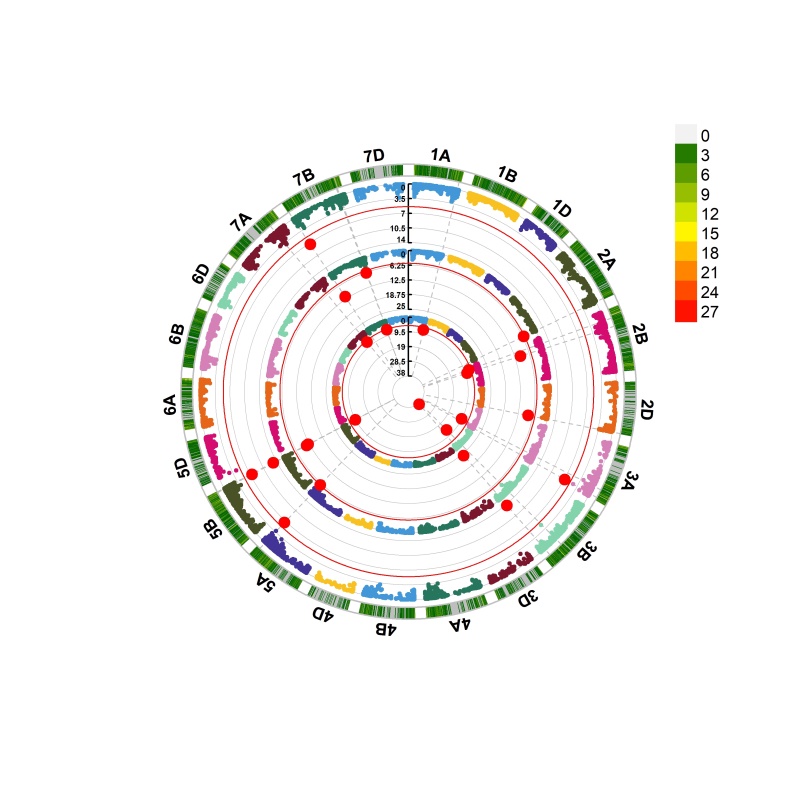

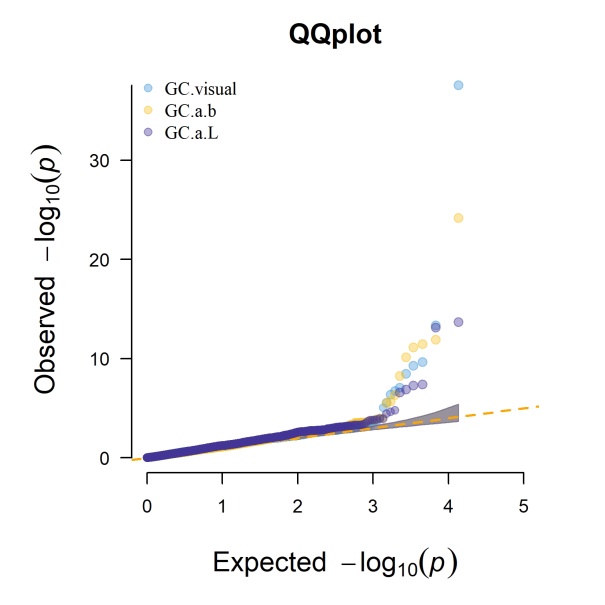
**

a

**
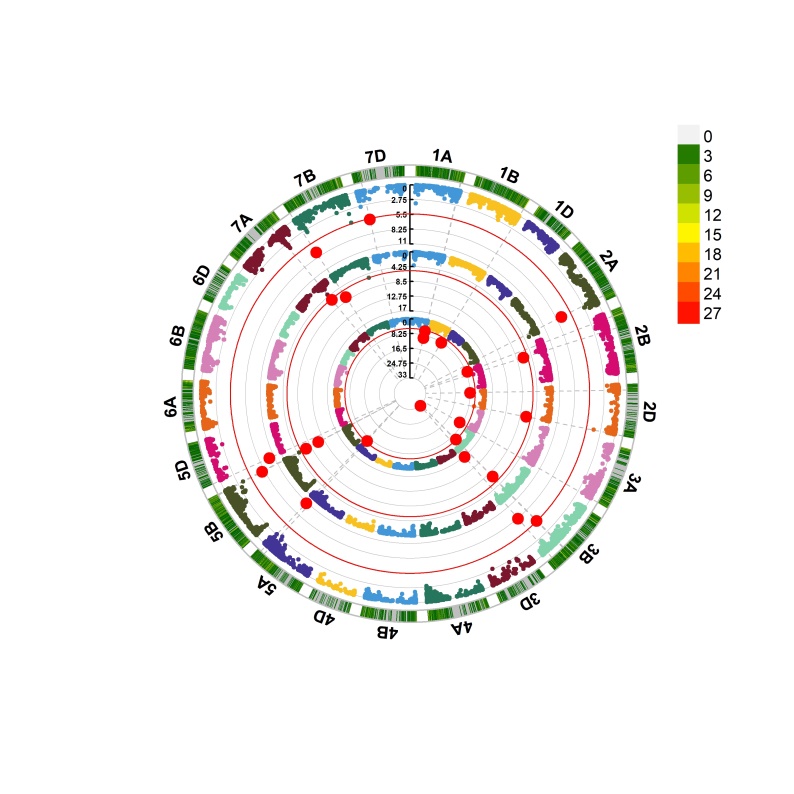

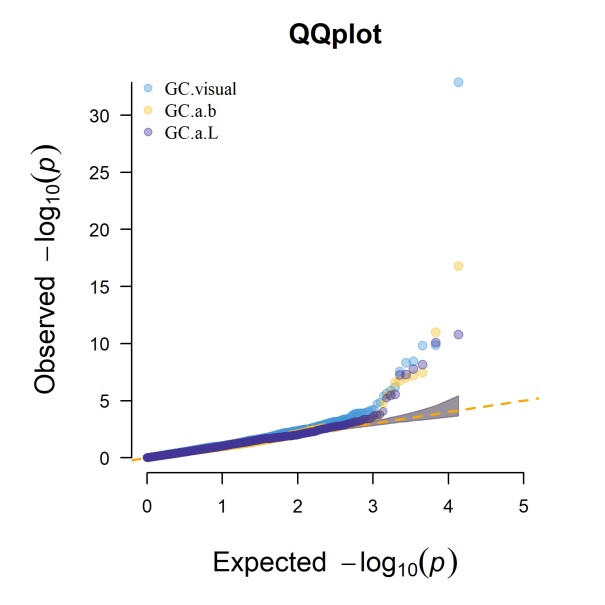
**

c

b

**
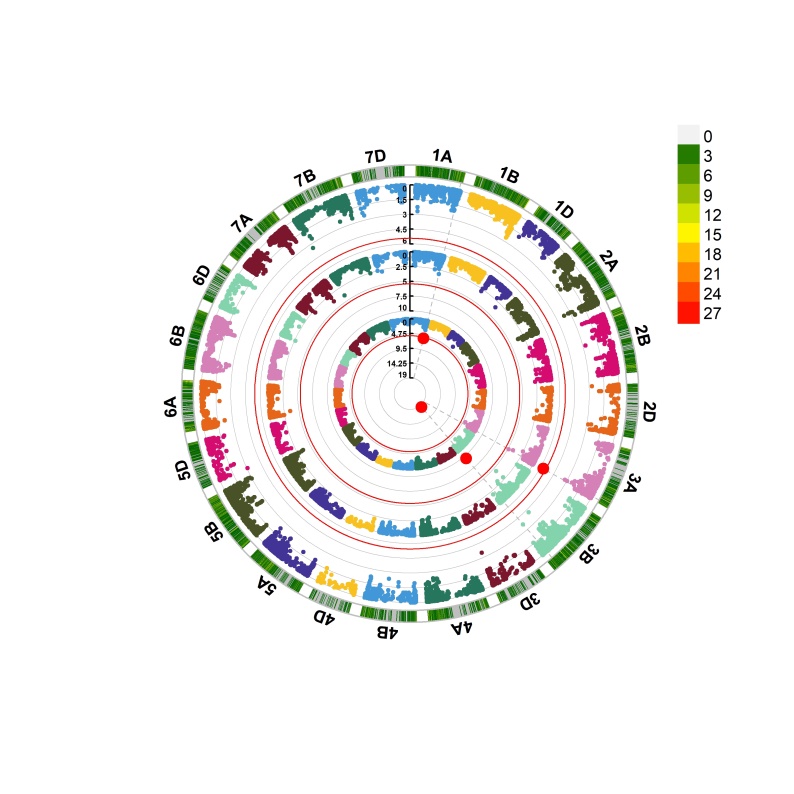

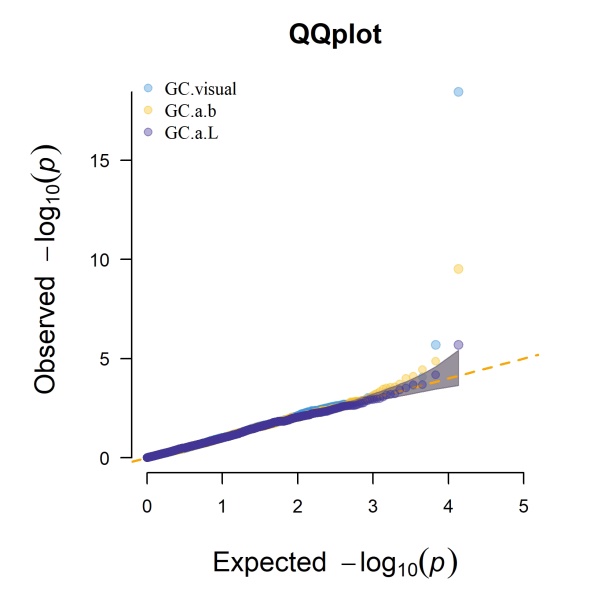
**

**
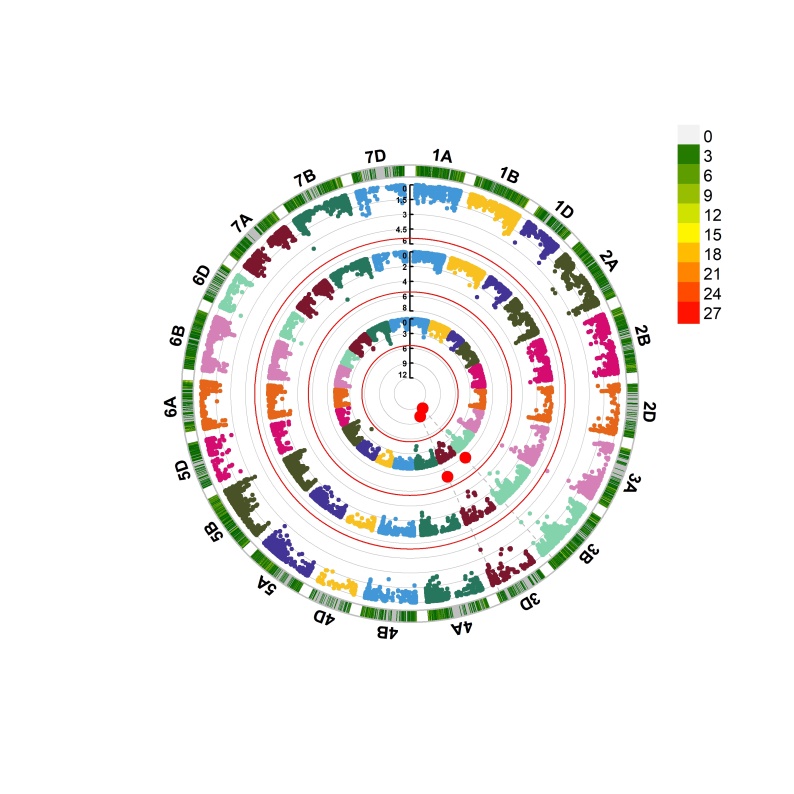

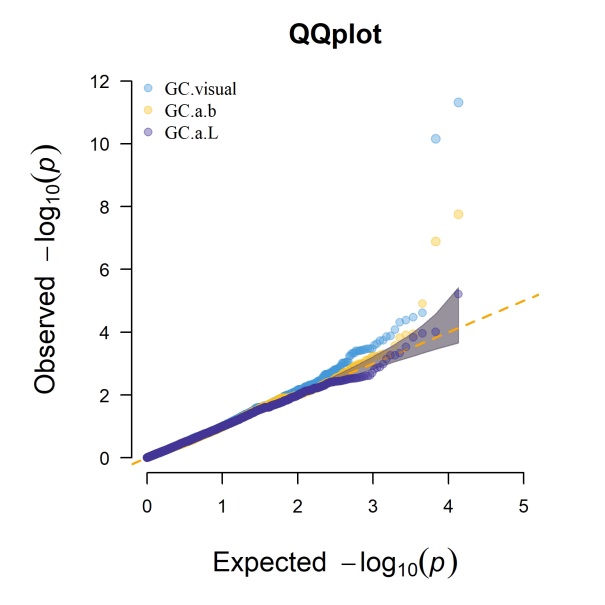
**

d

**
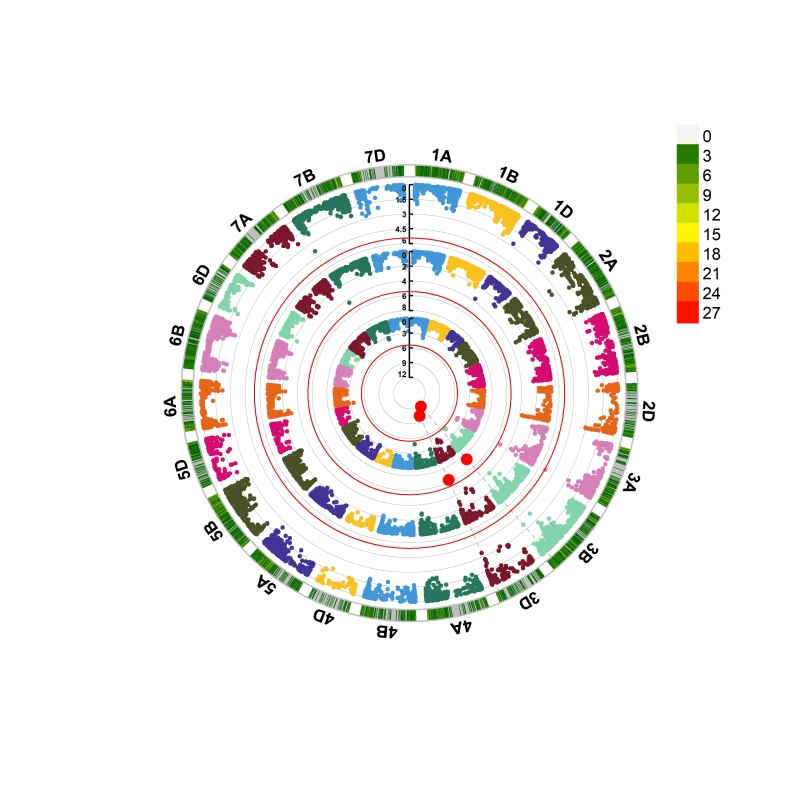

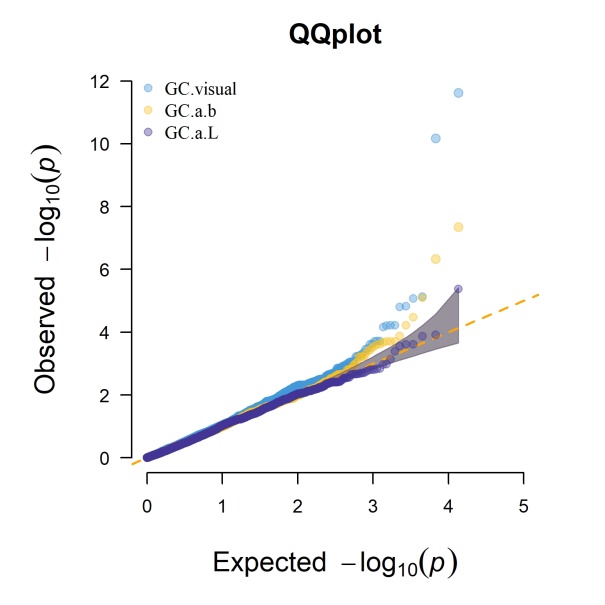
**

e

**Figure S5.** Manhattan plots and Q-Q plots for GC traits by using Blink (a), FarmCPU (b), MLMM (c), MLM (d) models in GAPIT and MLM (e) model in Tassel. From the inner circle to the outer circle in manhattan plots, these circles represent GC.visual, GC.a.b, GC.a.L traits. Red dots represent the significant markers in manhattan plots.


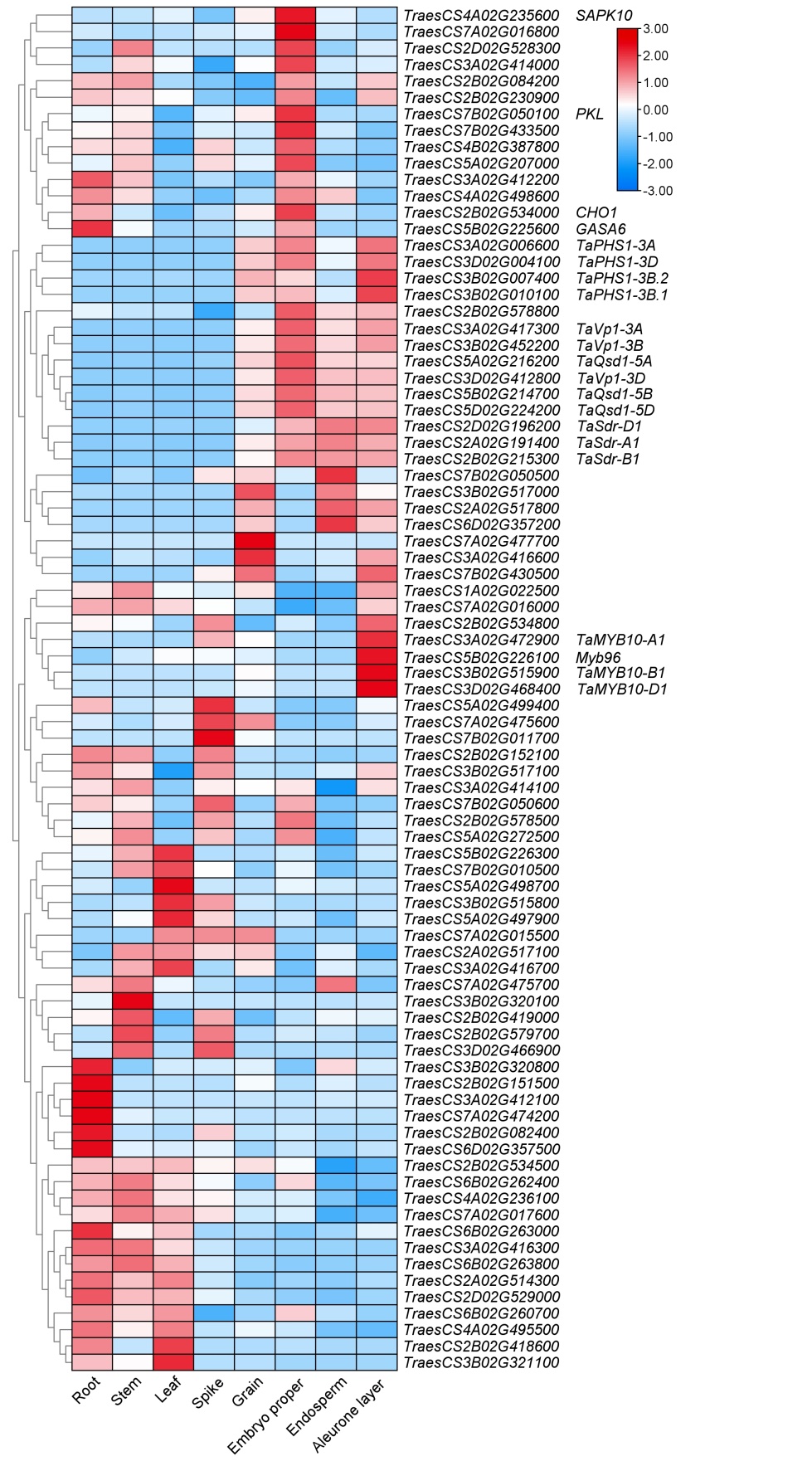


**Figure S6.** Heatmap representing the expression profile of genes and candidate genes related to PHS resistance. Gene expression values are expressed as log2 transformed TPM (transcripts per million) values and normalized by row scale.


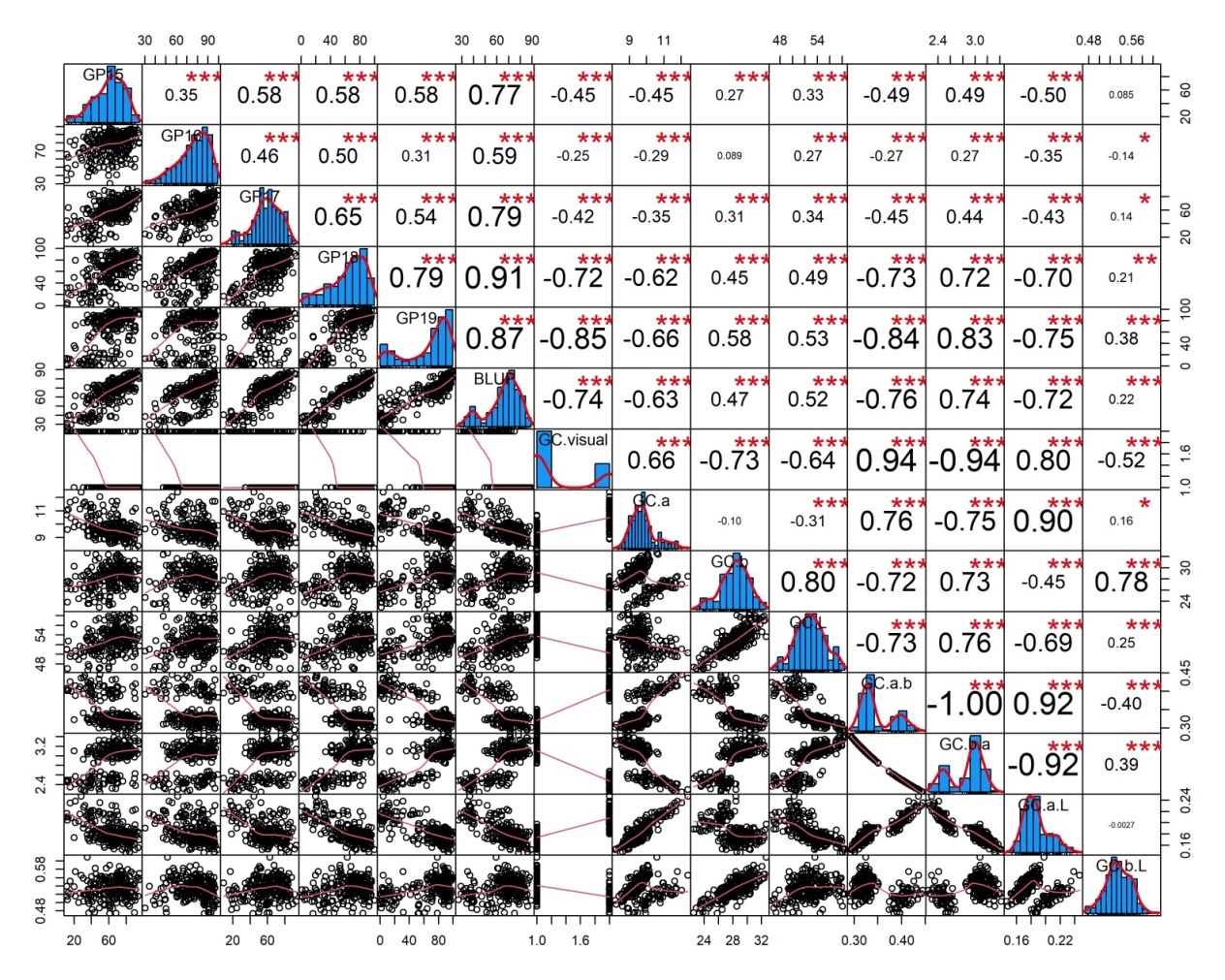


**Figure S7.** Correlation analysis between PHS resistance and GC in 235 wheat varieties. PHS resistance was evaluated using the spike-wet test, with germination percentage (GP) as the indicator. GP values from five growing seasons (2015–2019) were denoted as GP15, GP16, GP17, GP18, and GP19. The best linear unbiased prediction (BLUP) was calculated to represent PHS resistance across multiple environments. Grain color (GC) was assessed by visual inspection (GC.visual) and colorimeter measurements in the CIE Lab* color space, including GC.a (red-green axis), GC.b (yellow-blue axis), and GC.L (lightness). Derived ratios were calculated as GC.a.b (a/b), GC.b.a (b/a), GC.a.L (a/L), and GC.b.L (b/L). Significance levels of Pearson’s correlation coefficients: * = p<0.05, ** = p<0.01, and *** = p<0.001.
